# Ca^2+^ Complexation With Relevant Bioligands in Aqueous Solution: A Speciation Study With Implications for Biological Fluids

**DOI:** 10.3389/fchem.2021.640219

**Published:** 2021-02-24

**Authors:** Donatella Aiello, Federica Carnamucio, Massimiliano Cordaro, Claudia Foti, Anna Napoli, Ottavia Giuffrè

**Affiliations:** ^1^ Dipartimento di Chimica e Tecnologie Chimiche, Università Della Calabria, Arcavacata di Rende, Italy; ^2^ Dipartimento di Scienze Chimiche, Biologiche, Farmaceutiche Ed Ambientali, Università di Messina, Messina, Italy

**Keywords:** Ca^2+^, biological ligands, speciation in biological fluids, sequestration, potentiometry, ^1^H NMR spectroscopy, mass spectrometry, thermodynamic parameters

## Abstract

A speciation study on the interaction between Ca^2+^ and ligands of biological interest in aqueous solution is reported. The ligands under study are l-cysteine (*Cys*), d-penicillamine (*PSH*), reduced glutathione (*GSH*), and oxidized glutathione (*GSSG*). From the elaboration of the potentiometric experimental data the most likely speciation patterns obtained are characterized by only protonated species with a 1:1 metal to ligand ratio. In detail, two species, CaLH_2_ and CaLH, for systems containing *Cys*, *PSH*, and *GSH*, and five species, CaLH_5_, CaLH_4_, CaLH_3_, CaLH_2_, and CaLH, for system containing *GSSG*, were observed. The potentiometric titrations were performed at different temperatures (15 ≤ *t*/°C ≤ 37, at *I* = 0.15 mol L^−1^). The enthalpy and entropy change values were calculated for all systems, and the dependence of the formation constants of the complex species on the temperature was evaluated. ^1^H NMR spectroscopy, MALDI mass spectrometry, and tandem mass spectrometry (MS/MS) investigations on Ca^2+^-ligand solutions were also employed, confirming the interactions and underlining characteristic complexing behaviors of *Cys*, *PSH*, *GSH*, and *GSSG* toward Ca^2+^. The results of the analysis of ^1^H NMR experimental data are in full agreement with potentiometric ones in terms of speciation models and stability constants of the species. MALDI mass spectrometry and tandem mass spectrometry (MS/MS) analyses confirm the formation of Ca^2+^-L complex species and elucidate the mechanism of interaction. On the basis of speciation models, simulations of species formation under conditions of some biological fluids were reported. The sequestering ability of *Cys*, *PSH*, *GSH*, and *GSSG* toward Ca^2+^ was evaluated under different conditions of pH and temperature and under physiological condition.

## Introduction

Calcium is the fifth most important element in the human body. It is indispensable for life, for the regulation of metabolism and maintenance of structure (Peterlik and Stoeppler, 2004). It behaves like an intracellular “second messenger” in numerous processes, namely, neurotransmitter release, cellular proliferation and differentiation, and control of exocrine and endocrine secretions (Bringhurst and Potts., 1979; Broaudus, 1993). In human body, about 99% of total calcium (1.0–1.3 kg in adults) (Hluchan and Pomerantz, 2002) is found in the bones. The remaining part, 1%, is present in intra- and extracellular fluids. Free calcium concentration in the cell ranges between 10^−6^ and 10^−8^ mol kg^−1^. It is about 10^−3^ mol kg^−1^ in the sarcoplasm (Frausto da Silva and Williams, 2001a). The mean Ca^2+^ concentration in the plasma is 2.5 mmol L^−1^, of which about 50% is present as free ion; the remaining part is bound for 40% to plasma proteins and for 10% to citrate and phosphate. The rigid control of free calcium in the plasma is very crucial, as even small concentration changes can cause significant variations in the skeletal site, as well as intracellular free calcium, with harmful consequences for bone health (Peterlik and Stoeppler, 2004; Whedon, 1980). Calcium homeostasis is based on a dynamic equilibrium of its fluxes between three different body compartments, namely, extracellular fluid, intracellular one, and skeletal tissue. As regards the physiological role of calcium, it includes the control of many kinase reactions in metabolism, of dioxygen release in photosynthesis, and of dehydrogenases in oxidative phosphorylation (Frausto da Silva and Williams, 2001b). Ca^2+^ interacts preferably with oxygen donor groups. In the body fluids it can bind polymers, such as proteins, *via* carboxylate and phosphate sidechains. In the proteins, the main donor groups toward Ca^2+^ are represented by carboxylate and carbonyl centers (Frausto da Silva et al., 2001a).

*Cys* is one of the most important binding agents for metal cations in biological fluids (Laurie et al., 1979). Its concentration in normal human plasma is in the micromolar range (Brigham et al., 1960). The drug penicillamine, which has a very similar structure to *Cys*, was commonly employed in the treatment of Wilson’s disease (Walshe, 1956; Jones, 1991). *GSH* is a tripeptide consisting of the amino acids l-glutamic acid (Glu), *Cys*, and glycine (Gly). It exists in two forms: a reduced (*GSH*) and an oxidized one, i.e., dimer glutathione disulfide (*GSSG*) (Labib et al., 2016). *GSH* is ubiquitous antioxidant present in cells as well as in bacteria (Sies, 1999; Pompella et al., 2003; Kretzschmar et al., 2020; Meister and Anderson, 1983). In mammalian cells, concentrations greater than 12 mmol L^−1^ are reported (Dringen, 2000). Both *GSH* and its oxidized form, *GSSG*, are fundamental for the maintenance of the intracellular redox state (Shahid et al., 2020). They are considered biomarkers of oxidative stress in biological fluids as well as for the diagnosis of certain clinical disorders (Labib et al., 2016; Olmos Moya et al., 2017). The mechanism of antioxidant cellular defense *in vivo* is governed by *GSH*, oxidized continuously to disulfide glutathione (*GSSG*) (Davis and Hanumegowda, 2008). In healthy cells, the *GSH* form constitutes over 90% of glutathione (Labib et al., 2016). In addition to protecting cells from oxidative damage, *GSH* is involved in the complexation and transport reactions of metal ions (Olmos Moya et al., 2017). In blood, the normal values of *GSH* and *GSSG* are 3.8–5.5 and 0.2–0.5 μmol L^−1^, respectively (Labib et al., 2016).

**FIGURE 1:**
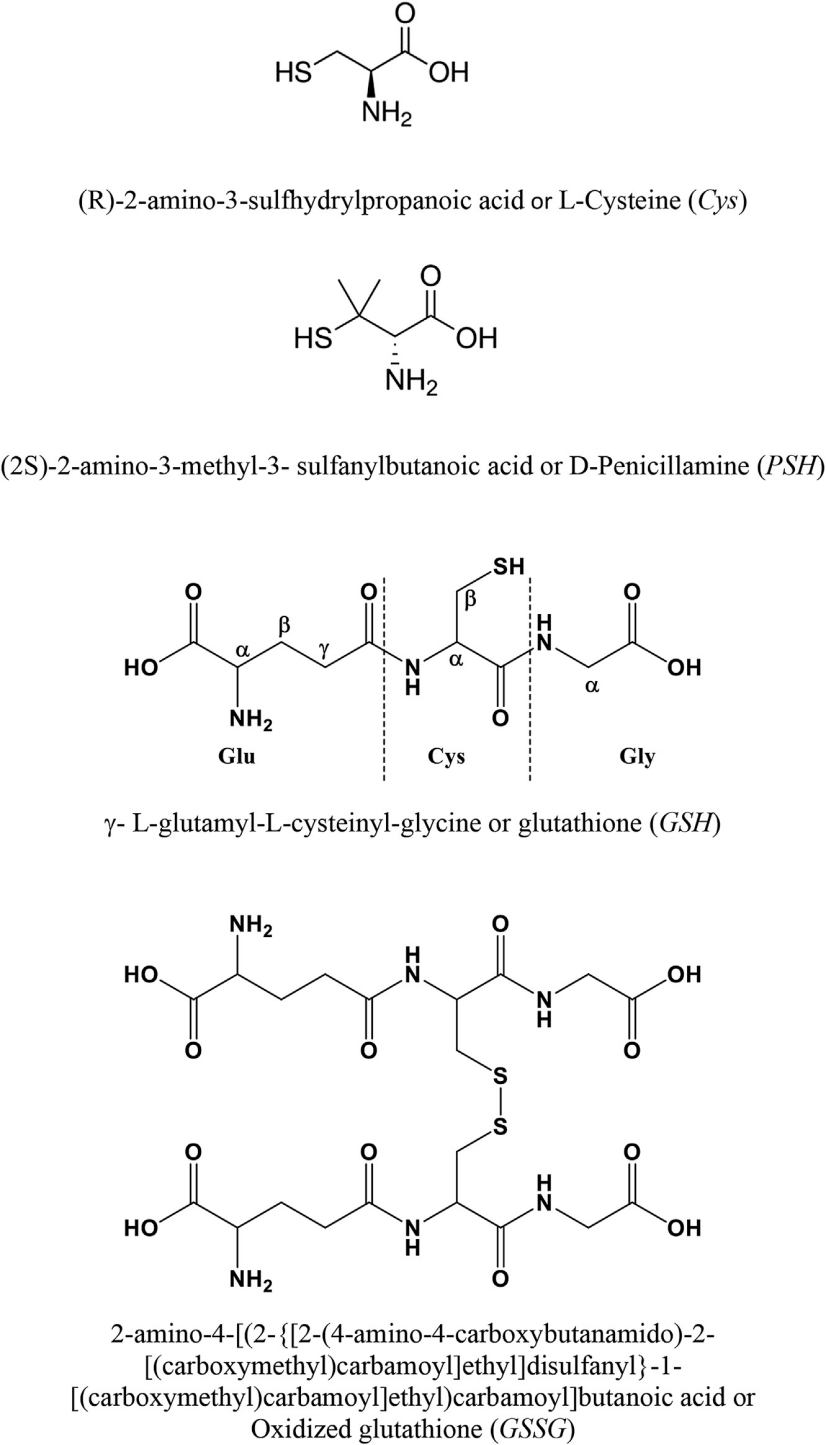
Ligands under study.

Given all these aspects, reliable assessment of the speciation of biologically relevant ligands with Ca^2+^ is crucial to understand and to model the behavior of these systems. Ligands under study are reported in Figure 1. In this study, the experimental measurements were performed by different techniques: potentiometry, ^1^H NMR spectroscopy, MALDI mass spectrometry, and MS/MS. The potentiometric titrations were carried out at different temperatures, 15 ≤ *t*/°C ≤ 37 and *I* = 0.15 mol L^−1^ in NaCl. Some simulations of species formation under conditions of biological fluids were reported. The sequestering ability of all ligands understudy toward Ca^2+^ was evaluated under different conditions of pH and temperature.

## Material and Methods

### Materials

The solutions containing calcium metal cation were obtained by weighing and dissolving the corresponding salt, calcium (II) chloride dihydrate (purity >99%, Fluka/Honeywell, Charlotte, North Carolina, US). Afterward calcium solutions were standardized by titration with EDTA (Ethylenediaminetetraacetic acid disodium salt, BioUltra, ≥99%, Sigma-Aldrich/Merck, Darmstadt, Germany) standard solution. Ligand solutions were prepared by weighing and dissolving, without further purification, the following products: l-cysteine (purity ≥99.5%, Fluka/Honeywell, Charlotte, North Carolina, US), d-penicillamine (purity ≥97%, Alfa-Aesar/Thermo Fisher, Kandel, Germany), reduced glutathione (purity ≥98%, Alfa-Aesar, Thermo Fisher, Kandel, Germany), and oxidized glutathione (purity 98%, Sigma-Aldrich/Merck, Darmstadt, Germany). The purity of the ligands was checked by alkalimetric titration. It was found to be greater than 99%. Solutions of hydrochloric acid and sodium hydroxide were obtained by dilution of Fluka (Fluka/Honeywell, Charlotte, North Carolina, US) ampoules and afterward they were standardized with sodium carbonate (≥99.5%, Sigma-Aldrich/Merck, Darmstadt, Germany) and potassium biphthalate (≥99.5%, Sigma-Aldrich/Merck, Darmstadt, Germany), respectively. Both salts were previously dried in an oven at 110 °C. Solutions of sodium hydroxide were reprepared very frequently and were kept in bottles with soda lime traps. Solutions of sodium chloride were obtained by weighing the corresponding salt (puriss., Sigma-Aldrich/Merck, Darmstadt, Germany), previously dried in an oven at 110 °C. Distilled water (conductivity <0.1 μS cm^−1^) and grade A glassware were employed for the preparation of all the solutions.

### Potentiometric Apparatus and Procedure

Two distinct systems were employed for the potentiometric titrations. In detail, the systems consist in an identical configuration consisting in an automatic dispenser Metrohm Dosino 800, a Metrohm model 809 Titrando potentiometer, and a Metrohm LL-Unitrode WOC combined glass electrode. Each potentiometric system was connected to a PC and the experimental titration data were acquired by the Metrohm TIAMO 2.2 software. It can control several parameters, such as e.m.f. stability, titrant delivery, and data acquisition. Estimated accuracy of this apparatus is ±0.15 mV and ±0.002 ml for e.m.f. and for readings of titrant volume, respectively.

Each titration consists in additions of volumes of NaOH standard to 25 ml of the solution containing Ca^2+^, ligand, and a supporting electrolyte (NaCl). Experimental details on potentiometric titrations are reported in Table 1. Glass jacket thermostated cells were employed for the measurements performed under different conditions of temperature (15 ≤ *t*/°C ≤ 37), by bubbling pure N_2_ in order to avoid CO_2_ and O_2_ inside the solutions and under magnetic stirring. For each measurement, an independent titration of HCl with standard NaOH was performed to calculate the standard electrode potential E^0^ and the pK_w_ value, under the same experimental ionic strength and temperature conditions.

**TABLE 1:** Experimental conditions for potentiometric and ^1^H NMR titrations at *I* = 0.15 mol L^−1^ in NaCl.

Technique	*t*/°C	C_M_/mmol L^−1^	C_L_/mmol L^−1^	M/L	pH range
Potentiometry	15–37	1–5	2–6	0.33–2	2–10
^1^H NMR	25	5–10	5–10	0.75–1.5	2–10.5
^1^H NMR	25	−	10^a^	−	2–10.5

^a^ Protonation measurements for GSSG ligand.

### NMR Apparatus and Procedure

The spectrometer employed for the collection of ^1^H NMR spectra is a Varian 500 F T-NMR. 1,4-Dioxane was used as internal reference (*δ*_CHdioxane_ = 3.70 ppm); the chemical shifts are referred to tetramethylsilane (TMS). All the measurements were carried out in a 9:1 H_2_O/D_2_O solution at t = 25 °C. Presaturation technique was employed to suppress the water signal. Experimental details on ^1^H NMR titrations are reported in Table 1.

### Mass Spectrometric Apparatus and Procedure

A water solution of 2 equivalents of each ligand (*Cys*, *PSH*, *GSH*, *GSSG*) was added dropwise to 1 mmol of CaCl_2_ dissolved in water with magnetic stirring for 2 h at room temperature. MALDI MS and MS/MS analyses were performed using a 5800 MALDI-TOF-TOF Analyzer (AB SCIEX) in reflection positive ion mode with a mass accuracy of 5 ppm. At least 5000 laser shots were typically accumulated with a laser pulse rate of 400 Hz and 1000 Hz in the MS and MS/MS mode, respectively. MS/MS experiments were performed using ambient air as collision gas with a medium pressure of 10^−6^ Torr and a collision energy of 1 kV, with a mass accuracy of 20 ppm. After acquisition, spectra were processed using Data Explorer version 4.0. MALDI MS and MS/MS experimental conditions were optimized using sinapinic acid (SA, 5 mg/ml in H_2_O/CH_3_CN 40:60, v/v; with 0.1% TFA) as matrix for all ligands. The sample loading was performed by dried droplet method for all ligands, spotting 1 μL of sample/matrix premixed solution (1:5, v/v ratio).

### Calculations

Experimental data of potentiometric titrations were processed using BSTAC and STACO programs. They allow for obtaining the best speciation model for each system under study, the formation constant values of the species, and the parameters of a titration (standard potential E^0^, analytical concentration of the reagents, and junction potential). The parameters for the dependence of complex formation constants on temperature were obtained by LIANA program. More details on software employed in the refinement of the experimental data are reported in De Stefano et al. (1997). For ^1^H-NMR titrations, HypNMR software was employed to obtain protonation and formation constant values, as well as the individual chemical shift of each species, using the observed signals and assuming fast mutual exchange in the NMR time scale (Frassineti et al., 1995). HySS program was used to obtain the speciation diagrams and the formation percentages of the complex species (Alderighi et al., 1999).

## Results and Discussion

In the calculations, protonation constants of ligands understudy (Cardiano et al., 2008; Crea et al., 2008; Cardiano et al., 2013) and hydrolytic constant of Ca^2+^ were taken into account. They are reported in Supplementary Tables S1 and S2.

Potentiometric measurements were carried out under different conditions of temperature and metal-ligand ratios, to choose the most appropriate speciation model and to be able to refine the formation constants of the species in solution. The formation constants of Ca^2+^(M)-ligand(L) species are expressed as overall formation constants (β) and stepwise formation constants (*K*). The reactions are the following (charges are omitted for simplicity):

$$\text{M}+\text{L}+\text{rH}=\text{MLH}\;{\text{β}}^{\text{MLHr}},$$ (1)

$${\text{M}+\text{LH}}_{\text{r}}{=\text{MLH}}_{\text{r}}\;K^{\text{MLHr}}.$$ (2)

Within the speciation studies, the most reliable model for a metal-ligand system is chosen by taking into account several factors, such as the simplicity of the model itself, the statistical parameters (standard and mean deviation on the fit), the variance ratio between the chosen model and others, and the formation percentages of the formed species (Filella, 2005).

### Speciation Profiles and Aqueous Behavior

**FIGURE 2:**
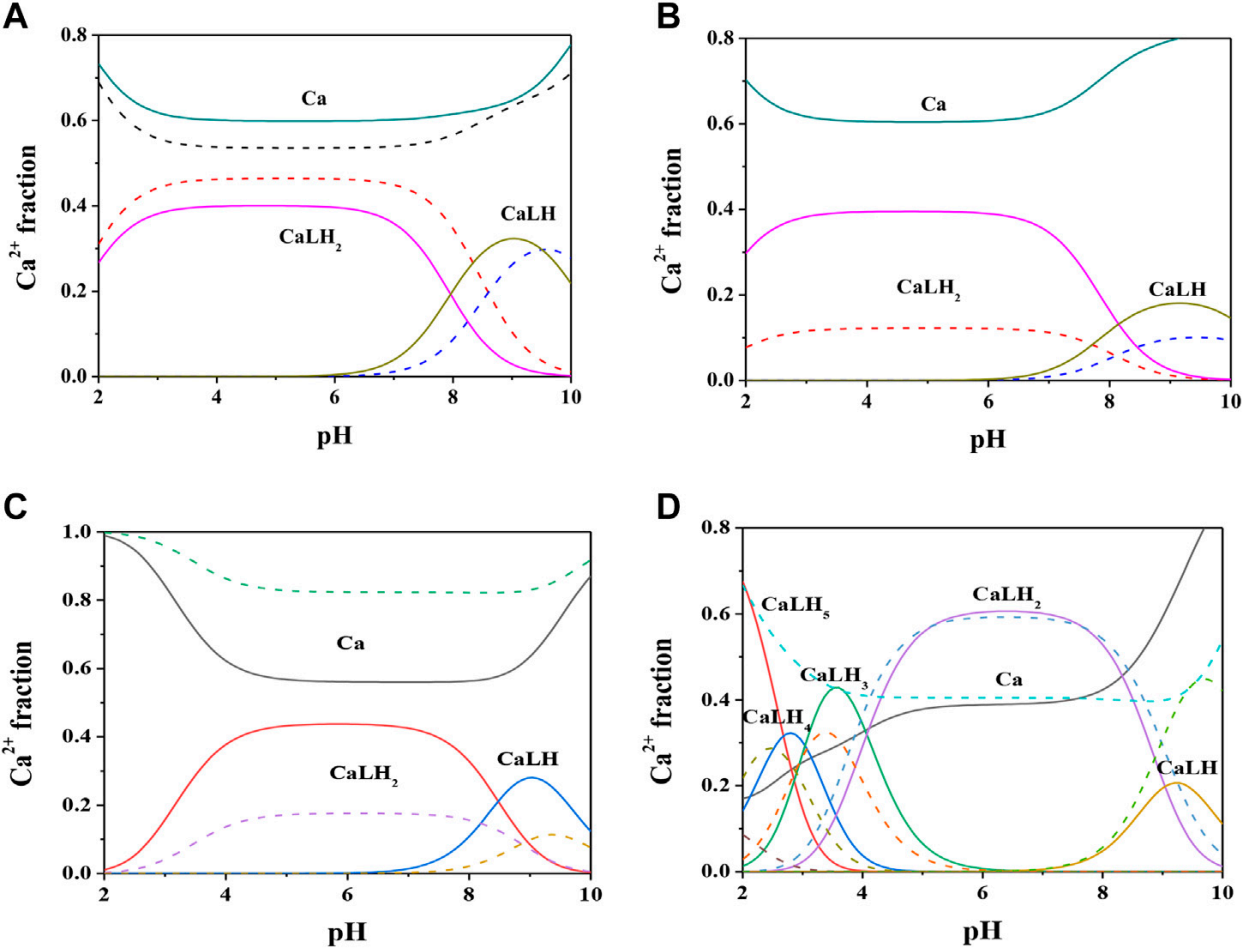
Speciation diagrams of Ca^2+^-ligand (L) systems at *t* = 15 °C (dotted lines) and *t* = 37 °C (solid lines), C_M_ = 2 mmol L^−1^, C_L_ = 4 mmol L^−1^, *I* = 0.15 mol L^−1^ in NaCl **(A)** L = *Cys* **(B)** L = *PSH* **(C)** L = *GSH* **(D)** L = *GSSG*.

Formation constant values of Ca^2+^-*Cys*, *PSH*, *GSH*, *GSSG* species obtained *via* potentiometric measurements at different temperatures and *I* = 0.15 mol L^−1^ were reported in Table 2. The speciation pattern for all the systems includes only 1:1 M:L species. *Cys*, *PSH*, and *GSH* show a very similar behavior with the same speciation model including only two significant species, namely, MLH_2_ and MLH. For all three systems, the stability of complex species in terms of stepwise formation constants is between a minimum of 1.57 (MLH species for Ca^2+^-*GSH* system, *t* = 25 °C) and a maximum of 3.66 (MLH_2_ species for Ca^2+^-*PSH* system, *t* = 37 °C). In Figure 2A the speciation diagram of Ca^2+^-*Cys* species is depicted at *I* = 0.15 mol L^−1^ and *t* = 15, 37 °C. Under physiological conditions (*t* = 37 °C, *I* = 0.15 mol L^−1^), MLH_2_ species is formed in the range 2 ≤ pH ≤ 9 and reaches a metal fraction of 0.4 in the range 3 ≤ pH ≤ 7. The main complex species in the range 8 ≤ pH ≤ 10 is MLH with a maximum metal fraction corresponding to 0.3 at pH = 9.5.

**TABLE 2:** Formation constants of Ca^2+^-*Cys*, *PSH*, *GSH*, *GSSG* species at different temperatures at *I* = 0.15 mol L^−1^ in NaCl.

Ligand	Species		logβ ^a^	
		*t* = 15 °C	*t* = 25 °C	*t* = 37 °C
*Cys*	MLH_2_	21.22 ± 0.01^b^	20.76 ± 0.03^b^	20.10 ± 0.06^b^
	MLH	12.62 ± 0.03	12.50 ± 0.04	12.14 ± 0.06
*PSH*	MLH_2_	20.86 ± 0.08	21.30 ± 0.08	21.65 ± 0.08
	MLH	13.00 ± 0.08	13.37 ± 0.09	13.89 ± 0.08
*GSH*	MLH_2_	20.39 ± 0.05	19.97 ± 0.03	20.14 ± 0.01
	MLH	11.53 ± 0.06	11.02 ± 0.07	11.66 ± 0.02
*GSSG*	MLH_5_	29.88 ± 0.09	30.18 ± 0.09	30.45 ± 0.06
	MLH_4_	28.28 ± 0.06	28.16 ± 0.09	27.77 ± 0.06
	MLH_3_	25.40 ± 0.06	25.12 ± 0.08	24.73 ± 0.06
	MLH_2_	21.64 ± 0.04	21.19 ± 0.07	20.66 ± 0.04
	MLH	12.66 ± 0.03	12.15 ± 0.08	11.62 ± 0.05
			log*K* ^c^	
*Cys*	MLH_2_	2.45	2.46	2.32
	MLH	2.20	2.34	2.26
*PSH*	MLH_2_	2.07	2.89	3.66
	MLH	2.25	2.83	3.58
*GSH*	MLH_2_	1.77	1.89	2.41
	MLH	1.81	1.57	2.41
*GSSG*	MLH_5_	1.90	2.71	3.56
	MLH_4_	2.48	2.84	2.99
	MLH_3_	2.71	2.91	3.05
	MLH_2_	2.72	2.75	2.75
	MLH	2.80	2.53	2.27

^a^ Overall formation constants.

^b^ ≥95% of confidence interval.

^c^ Stepwise formation constants.

Formation constants of Ca^2+^-*PSH* species are quite higher with respect to Ca^2+^-*Cys* ones. For example, stepwise formation constant values at *t* = 37 °C resulted between 3.58 and 3.66. The differences between stepwise formation constants of species formed by *PSH* and *Cys* with Ca^2+^, under physiological conditions, are Δlog*K =* 1.3 for both MLH_2_ and MLH. The speciation diagram, represented in Figure 2B, refers to Ca^2+^-*PSH* system, under physiological conditions. MLH_2_ species is formed in the wide interval 2 ≤ pH ≤ 8, reaching a maximum metal fraction of 0.4; MLH species is present in the range 8 ≤ pH ≤ 10 with a lower metal fraction (0.2).

For Ca^2+^-*GSH* system, stepwise formation constant values at *t* = 37 °C are equal to 2.41 for both species. The differences between formation constants of Ca^2+^-*PSH* and -*GSH* species, under physiological conditions are Δlog*K =* 1.2 for both. The speciation diagram, depicted in Figure 2C, refers to Ca^2+^-*GSH* system, under physiological conditions. It shows that MLH_2_ is the main complex species in the wide interval 2 ≤ pH ≤ 9, with a maximum metal fraction of 0.4, and MLH predominates in the range 8.5 ≤ pH ≤ 10, with a metal fraction of 0.3.

A separate discussion must be made for the system containing *GSSG*. As expected from the presence of the numerous protonable groups on molecule, the speciation model is very rich in complex species, namely, MLH_5_, MLH_4_, MLH_3_, MLH_2_, and MLH. Their stability is comparable to the values found for the other ligands already discussed. As an example, at *t* = 37 °C and *I* = 0.15 mol L^−1^, log*K* values, referring to stepwise formation constants, range between 2.27 and 3.56 for the five species. Speciation profile for Ca^2+^-*GSSG* system is represented in Figure 2D. Under physiological conditions, the less significant species is MLH one, while the most significant species is MLH_2_, which predominates in the pH range between 4.5 and 9 reaching metal fraction of 0.6. The most protonated species, MLH_5_, MLH_4_, and MLH_3_, predominant at pH < 4.5, reach maximum metal fractions equal to 0.65, 0.3, and 0.45, respectively. MLH species is significant only at pH > 9, with a metal fraction of 0.2.

### ^1^H NMR Spectroscopy

The interaction of ligands of biological interest with metal cations in aqueous solution had been already studied by our research group with several spectroscopic techniques, such as ^1^H NMR (Cardiano et al., 2008; Cardiano et al., 2011; Cardiano et al., 2013; Cardiano et al., 2016), UV-Vis (Falcone et al., 2011; De Stefano et al., 2014), Mössbauer (Cardiano et al., 2006), and Raman (Cassone et al., 2019). In the literature, there are some recent papers that report ^1^H NMR investigations on *GSSH* and *PSH* with metal cations other than Ca^2+^ (Sisombath et al., 2014; Kretzschmar et al., 2020). More in detail, Sisombath *et al.* report a complexation study on Pb^2+^ with *PSH* by ^1^H NMR analysis, in D_2_O solutions at pH = 9.6, at various M:L molar ratios. The data here reported are comparable with that reference and specifically it is possible to underline the same trend relative to the significant chemical shift of CH-2 (Δδ = 0.6 ppm) and of only one of the two -CH_3_.

**FIGURE 3:**
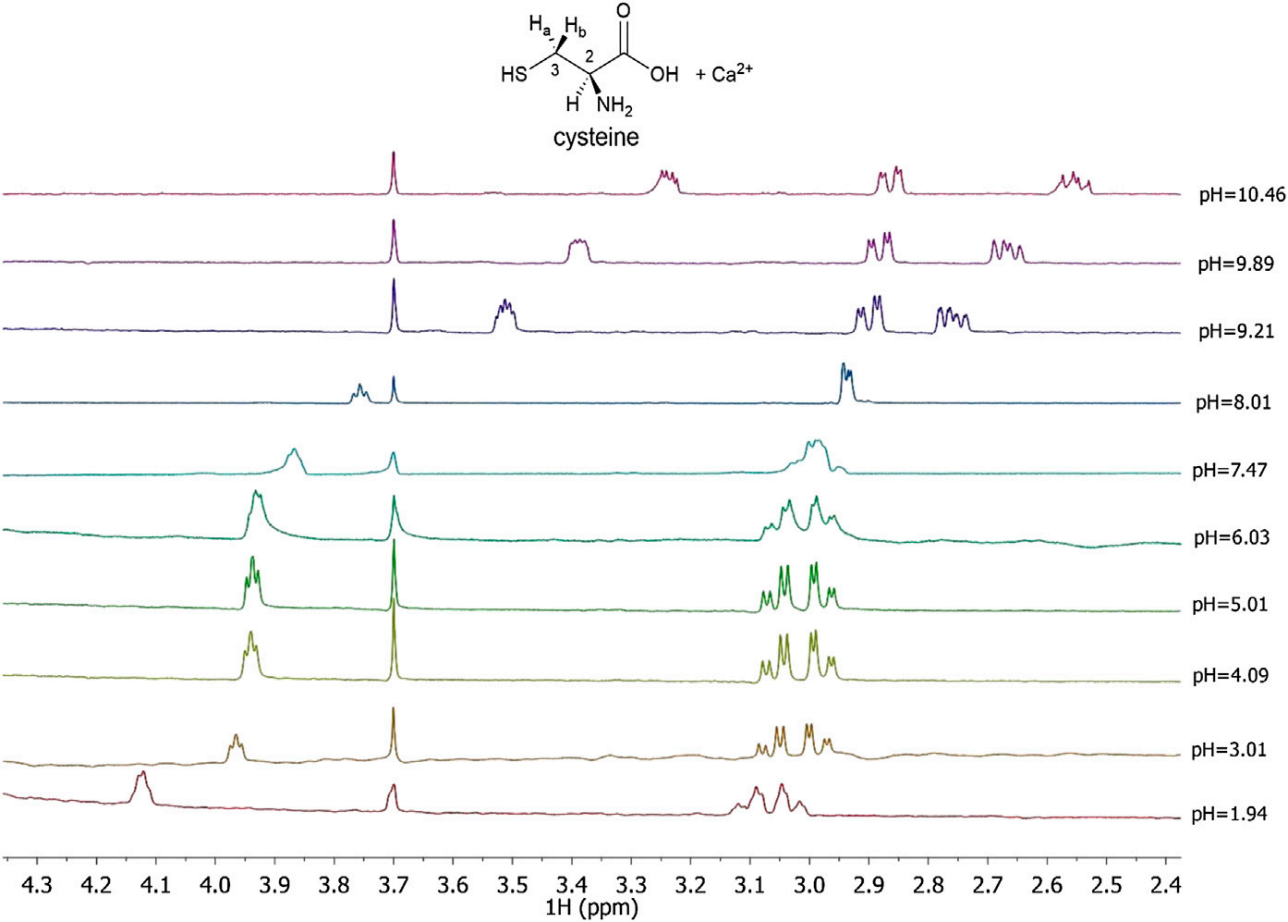
^1^H NMR spectra on solutions containing Ca^2+^ (M) and *Cys*(L) at C_M_ = 7.5 mmol L^−1^, C_L_ = 10 mmol L^−1^, *t* = 25 °C, *I* = 0.15 mol L^−1^ in NaCl, 1.94 ≤ pH ≤ 10.46.

In this paper ^1^H NMR spectra of Ca^2+^-*Cys* species, reported in Figure 3 at different pH values and *t* = 25 °C, show a chemical shift of the signals related to the proton in 2 and to the two protons in 3, indicated as H-2, H_a_-3, and H_b_-3, respectively for *Cys*. At pH < 8 there is a triplet for H-2 and a double doublet (*dd*) for H_a_-3 and H_b_-3. At pH > 8, the complexity of the signals increases wherein a multiplet for H-2, a *dd* for H_a_-3, and a *dd* for H_b_-3 are shown. The chemical shift of the H-2 proton to the increase of pH is approximately 0.8 ppm upfield due to the increase in the negative charge for the deprotonation of the carboxyl group and subsequently of the thiol group. A similar trend is evident for protons in 3; in this case the chemical shift is about 0.3 ppm. Much more interesting is the splitting of the signals into two different *dd*, which can be interpreted with greater rigidity of the ligand for the presence of a dianion or for the interaction with the metal cation as well. This AMX system is therefore due to the different magnetic properties of the two protons in 3 and consequent more complex coupling between the three protons H-2, H_a_-3, and H_b_-3. The interaction with Ca^2+^ is evident from the comparison with the corresponding chemical shift values of *Cys* alone, under the same experimental conditions.

^1^H NMR spectra of Ca^2+^-*PSH*, Ca^2+^-*GSH* solutions were reported in Supplementary Figures S2-S3. Both ligands evidenced a similar behavior to *Cys*, as their spectra NMR showed a significant shift in signals at the change of pH. The interaction of each ligand with Ca^2+^ is highlighted by the comparison with the corresponding chemical shift values of the ligand in the absence of the metal cation under the same experimental conditions. The comparison of the formation constant values obtained *via* potentiometric and ^1^H NMR measurements (see Table 3) shows satisfactory correspondence.

**TABLE 3:** Comparison between the experimental formation constants of Ca^2+^− ligand species and protonation constants of *GSSG* obtained *via* ^1^H NMR and potentiometry at *t* = 25 °C and *I* = 0.15 mol L^−1^.

Ligand	Species		logβ^a^
		^1^H NMR	Potentiometry
*Cys*	MLH_2_	20.4 (2)^b^	20.76
	MLH	12.4 (1)	12.50
*PSH*	MLH_2_	21.0 (2)^b^	20.60
	MLH	12.15	12.15
*GSH*	MLH_2_	20.49 (8)^b^	19.97
	MLH	10.9 (3)	11.02
*GSSG*	LH	9.3 (2)^b^	9.618
	LH_2_	18.35 (9)	18.442
	LH_3_	22.22 (4)	22.212
	LH_4_	25.43 (6)	25.319
	LH_5_	27.467	27.467
	LH_6_	28.987	28.987
	MLH_5_	30.18	30.18
	MLH_4_	28.16	28.16
	MLH_3_	25.12	25.12
	MLH_2_	21.21 (6)^b^	21.19
	MLH	12.04 (7)	12.15

^a^ Overall formation constants.

^b^ ≥95% of confidence interval.

**FIGURE 4:**
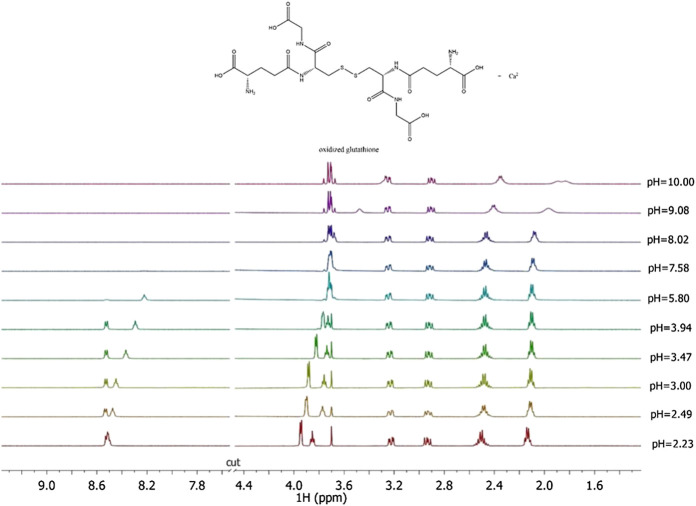
^1^H NMR spectra on solutions containing Ca^2+^ (M) and *GSSG*(L) at C_M_ = 8 mmol L^−1^, C_L_ = 6 mmol L^−1^, *t* = 25  °C, *I* = 0.15 mol L^−1^ in NaCl, 2.23 ≤ pH ≤ 10.00.

Here the NMR analysis of the free *GSSG* ligand at different pH values is reported. ^1^H NMR spectra of *GSSG* in 10% D_2_O/H_2_O solution show only six signals due to the symmetry to the S-S bond. Table 3 shows the comparison between the protonation constant values obtained by potentiometric and ^1^H NMR measurements. It was possible to obtain the values relating to the first four protonation constants (LH, LH_2_, LH_3_, and LH_4_), while those relating to the LH_5_ and LH_6_ species were kept constant using the values obtained by potentiometry. The agreement among the results obtained by the two different techniques was excellent. ^1^H NMR spectra registered on Ca^2+^-*GSSG* solutions at *t* = 25 °C and *I* = 0.15 mol L^−1^, represented in Figure 4, show substantially the same signal pattern observed in the spectra relating to the solutions containing *GSSG* ligand (Supplementary Figure S4). In detail, at pH = 2.2 are present amide protons 4 and 13 at *δ* = 8.5 ppm (2 singlets), proton in 3 at 3.95 ppm (quartet), proton in 11 at *δ* = 3.85 ppm (multiplet), proton in 14 at *δ* = 3.22 ppm (multiplet), proton in 2 at *δ* = 2.93 ppm (dd), proton in 9 at *δ* = 2.50 ppm (multiplet), and proton in 10 at *δ* = 2.13 ppm (multiplet).

**FIGURE 5:**
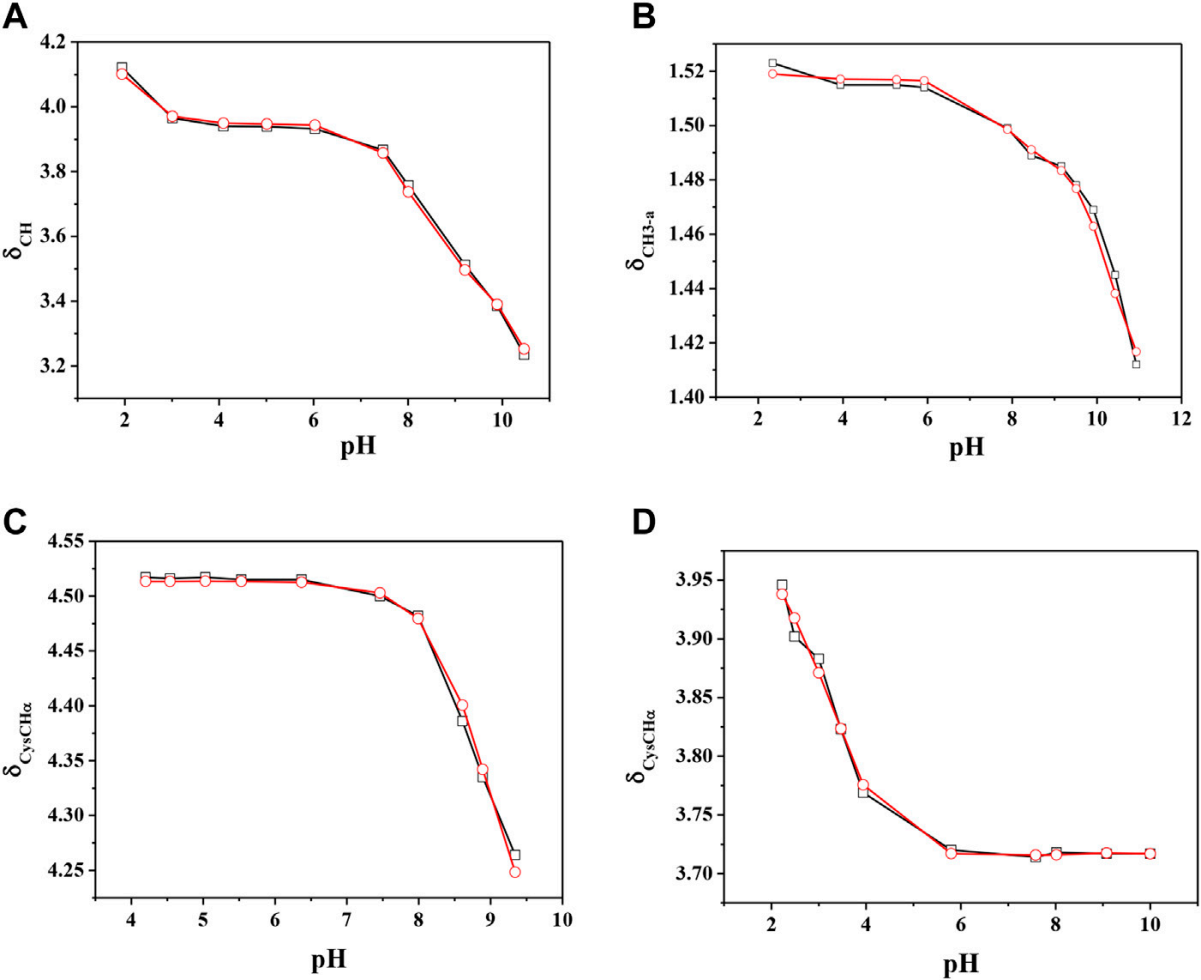
Experimental (□) and calculated (O) chemical shift values (in ppm) *vs*. pH of **(A)** CH on Ca^2+^(M)-*Cys*(L) solutions, **(B)** CH_3-a_ on Ca^2+^(M)-*PSH*(L) solutions, **(C)** CysCHα on Ca^2+^(M)-*GSH*(L) solutions, **(C)** CysCHα on Ca^2+^(M)-*GSSG*(L) solutions.

The chemical shift values of the individual species were calculated on the basis of formation percentages of each species in solution. These chemical shifts, reported in Supplementary Table S3, were used to determine the values of the formation constants of the complex species. In Table 3 these formation constant values obtained by ^1^H NMR titrations were reported, together with potentiometric ones. It is possible to notice a good agreement among the values determined by the two different techniques. For Ca^2+^-*GSSG* species only the values referring to MLH_2_ and MLH species were refined, keeping constant ones obtained by potentiometry related to MLH_5_, MLH_4_, and MLH_3_ species. The speciation model considered for all the systems is also confirmed by the complete overlap of the experimental and calculated chemical shift values shown in Figure 5. It should be noted that, at pH > 8, ^1^H NMR spectra on the solutions containing ligands in the presence of Ca^2+^ show significant differences with respect to the corresponding free ligands. At pH > 8, differences of Δδ between 0.05 and 0.10 ppm were calculated on average for all ligands, except for *GSSG*. From this experimental evidence, it can be assumed that *Cys*, *PSH*, *GSH*, and *GSSG* could behave as divalent ligands, binding Ca^2+^ and giving rise to cyclic complexes.

### MALDI MS and MS/MS

**FIGURE 6:**
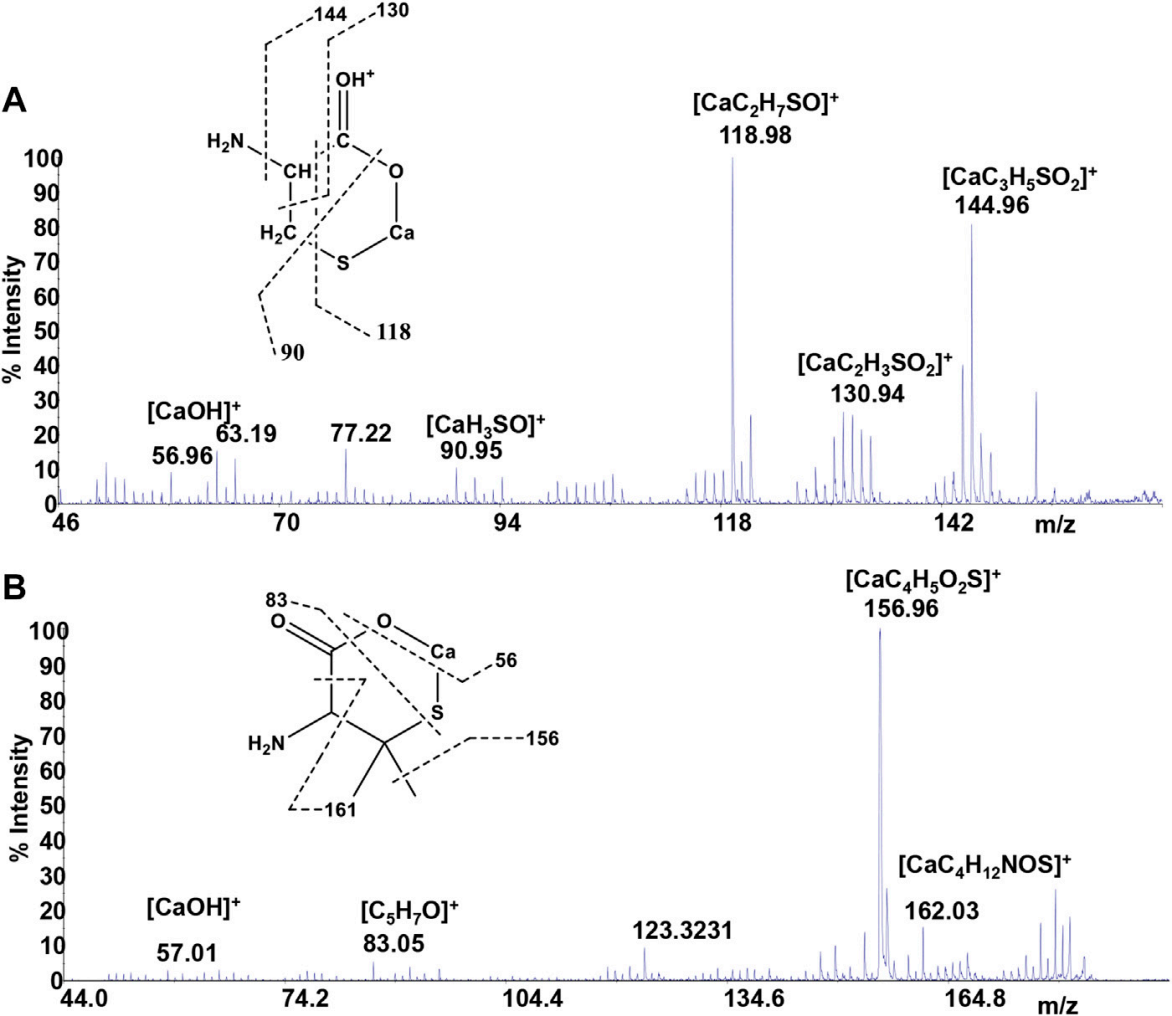
MS/MS spectrum of **(A)** *Cys* and **(B)** *PSH* in the presence of Ca^2+^.

Mass spectrometry combined with soft ionization methods as electrospray ionization (ESI) and matrix assisted laser desorption ionization (MALDI) is currently becoming a strategic approach to clarify structures and coordination sites in compounds where metals are chelated by biological ligands (Cardiano et al., 2009; Furia et al., 2014; Aiello et al., 2017; Aiello et al., 2018a; Chillè et al., 2020). MALDI-TOF/TOF-MS platforms can be used for the highly sensitive analysis of low molecular weight compounds (Aiello et al., 2020a) in complex matrices (Aiello et al., 2018b; Aiello et al., 2020b; Imbrogno et al., 2019; Salvatore et al., 2020). In order to investigate whether calcium binding by *Cys*, *PSH*, *GSH*, and *GSSG* induces formation of complexes, a water solution of 2 equivalents of each ligand was added dropwise to 1 equivalent of CaCl_2_ and complex association was analyzed by MALDI MS using sinapinic acid as matrix. Signals corresponding to complex ML with 1:1 stoichiometry are the most intense signals in the spectrum for all investigated systems. The molecular masses derived from these measurements are in good agreement with the calculated mass (within 5 ppm, Table 4). The simplest systems, represented by Ca^2+^-*Cys* and Ca^2+^-*PSH*, will briefly be discussed. Both ligands hold multiple donor sites that are capable of intramolecular stabilization of the metal-ligand species. The carboxylic acids, bearing donor groups in their α or β positions, generally act as bidentate ligands giving rise to cyclic structures (Aiello et al., 2018a; Falcone et al., 2013). Accordingly, the formation of [MLH]^+^ species suggests that *Cys* and *PSH* act as bidentate ligands giving rise to six-membered cycles (Figure 6). The simplicity of the MS/MS spectra suggests that only few fragmentation pathways are allowed for the decomposition of complexes. MALDI MS/MS spectrum of the system Ca^2+^-*Cys* (Figure 6A) reveals that the main fragmentation pathways of the precursor [MLH]^+^ (m/z 159.97, [CaC_3_H_6_NSO_2_]^+^) consist in the loss of low molecular species such as NH_2_ (m/z 144.96 [CaC_3_H_5_SO_2_]^+^) and CH_2_NH (m/z 130.95 ([CaC_2_H_3_SO_2_]^+^). However, some characteristic fragment ions can be found and correlate with the proposed structure. In particular, the formation of the ions of m/z 130.95 ([CaC_2_H_3_SO_2_]^+^), m/z 118.95 ([CaC_2_H_7_SO]^+^), and m/z 90.95 ([CaH_3_SO]^+^) arises from across ring fragmentation of a six-membered structure. Analogously, *PSH* leads to a cyclic structure ([MLH]^+^ of m/z 188.01 ([CaC_5_H_10_NO_2_S]^+^). Several distinguishing ion products were detected in the MS/MS spectra; all the peak assignments are described in Table 4 and Figure 6B. In agreement with the NMR data, it can be reasonably stated that *Cys* and *PSH* act as divalent ligands and that they bind the Ca^2+^ ion through O and S giving rise to six-membered cyclic complexes, as already observed for other ligands containing carboxylic and thiol groups (Cardiano et al., 2009).

**TABLE 4:** Mass spectrometry data of Ca^2+^-L species, reported as m/z values, formula assignments, and MS/MS values for fragment ions.

	Formula	m/z	Δppm
[(M-*Cys*)H]^+^	[CaC_3_H_6_NSO_2_]^+^	159.97	5.0
	[CaC_3_H_5_SO_2_]^+^	144.96	4.5
	[CaC_2_H_3_SO_2_]^+^	130.95	6.0
	[CaCH_3_SO_2_]^+^	118.95	5.3
	[CaH_3_SO]+	90.95	4.8
	[CaOH]^+^	56.97	4.0
[(M-*PSH*)H]^+^	[CaC_5_H_10_NO_2_S]^+^	188.01	6.0
	[CaC_4_H_5_O_2_S]^+^	156.96	4.5
	[CaC_4_H_9_O_2_S]^+^	161.00	5.5
	[C_5_H_7_O]^+^	83.05	5.3
	[CaOH]^+^	56.97	4.8
[(M-*GSH*)H]^+^	[CaC_10_H_16_N_3_SO_6_]^+^	346.04	5.0
	[CaC_10_H_16_N_3_SO_5_]^+^	330.04	4.5
	[CaC_10_H_14_N_3_O_6_]^+^	312.05	5.5
	[CaC_9_H_16_N_3_SO_4_]^+^	302.05	5.1
	[CaC_8_H_11_N_2_SO_4_]^+^	271.01	4.8
	[CaC_8_H_13_N_2_SO_3_]^+^	219.05	4.7
	[C_6_H_11_N_2_O_3_S]^+^	191.04	4.1
	[C_3_H_4_NO_2_]^+^	125.98	3.9
	[CaOH]^+^	56.97	5.3
[(M-*GSSG*)H]^+^	[CaC_20_H_31_N_6_O_12_S_2_]+	651.10	4.0
	[CaC_20_H_31_N_6_S_2_O_11_]^+^	635.60	4.2
	[CaC_20_H_31_N_6_S_2_O_10_]^+^	619.76	4.8
	[CaC_18_H_25_N_5_S_2_O_10_]^+^	576.07	5.2
	[CaC_15_H_24_N_5_S_2_O_5_]^+^	522.06	4.1
	[CaC_12_H_19_N_4_S_2_O_6_]^+^	379.07	4.7
	[CaC_10_H_12_N_3_SO_6_]^+^	346.04	5.3
	[CaC_10_H_14_N_3_O_6_]^+^	312.05	5.5
	[CaC_8_H_11_N_2_SO_4_]^+^	271.01	4.8
	[C_3_H_4_NO_2_]^+^	125.98	3.9

**FIGURE 7:**
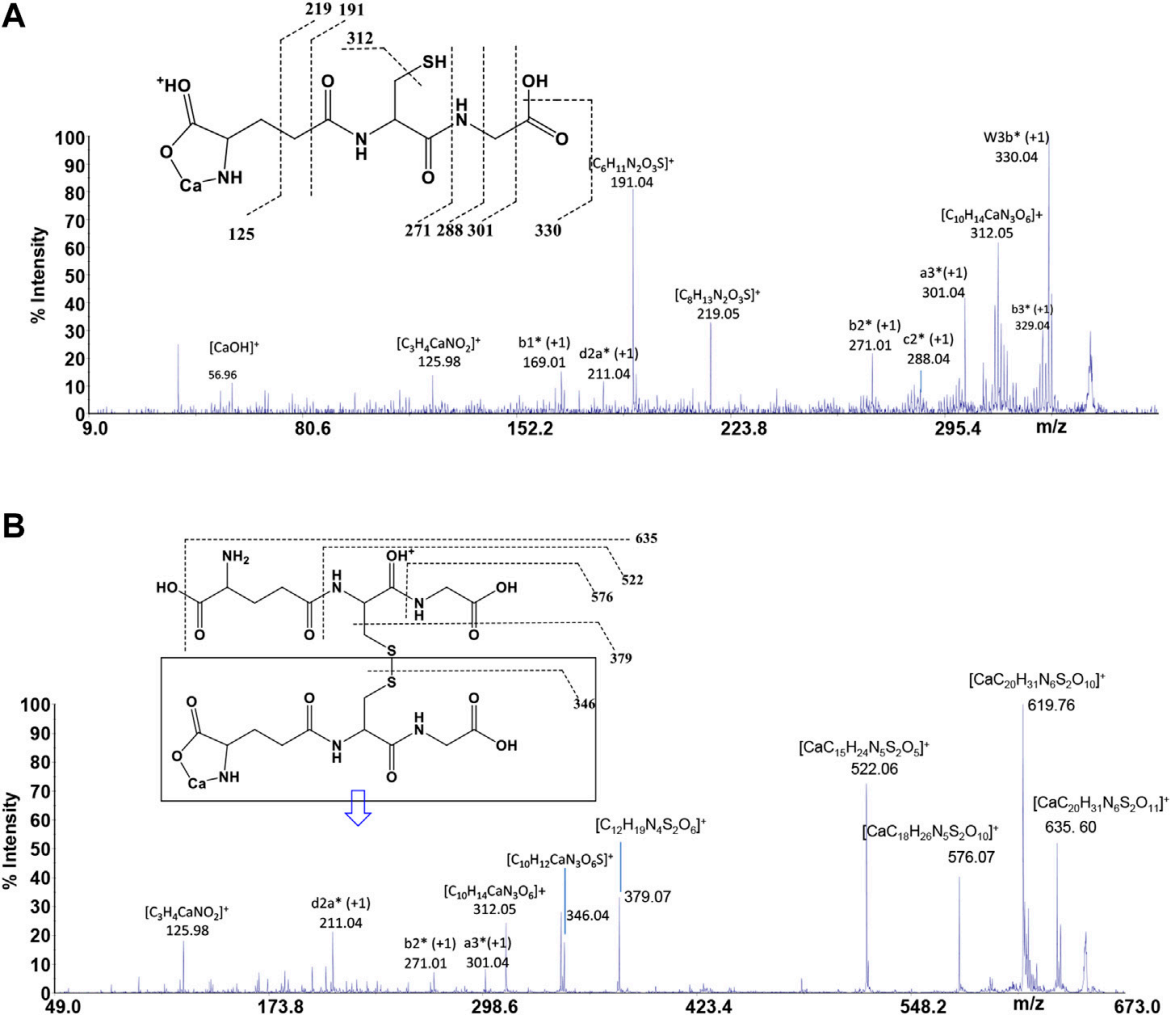
MS/MS spectrum of **(A)** *GSH* and **(B)** *GSSG* in the presence of Ca^2+^.

*GSH* is a tripeptide bearing two free -COOH groups, a -NH_2_ group, and a -SH group; it provides a hydrophilic interface and a handle for further reactivity with other functional molecules as well as metal ions. The metal coordination ability of *GSH* is well documented, highlighting its multichelating nature. The speciation of both reduced and oxidized forms of *GSH* in MS/MS condition was considered. Information about molecular mass of the Ca^2+^-*GSH* as well as Ca^2+^-*GSSG* complex is easily obtained using 1:1 Ca^2+^-*GSH* molar ratios. The peak at m/z 346.04 corresponds to the ion [MLH]^+^ in which *GSH* is deprotonated (i.e., *GSH* ^2-^) and therefore presumably bound to Ca^2+^ *via* -COOH and -NH amino groups. The calcium complex of *GSH* (m/z 346.04 [CaC_10_H_16_N_3_SO_6_]^+^) decomposes to give, besides major H_2_O and CO_2_ and H_2_S losses, small abundances of w_3b_*, a_3_*, b_2_*, c_2_*, b_1_*, and d_2a_* calcium containing and z_1_ non-calcium product ions (Figure 7A). Product ions, which contain the C terminus, are formed by losses of residues comprised of only one amino acid, suggesting that the primary binding site for the Ca^2+^ is the N terminus of the peptide. The formation of the ion of m/z 125.98 ([C_3_H_4_CaNO_2_]^+^) and its counterpart m/z 219.05 [C_8_H_13_N_2_O_3_S]^+^ indicates that *Glu* is calcium-binding amino acid. The Ca^2+^-*GSSG* (m/z 651.10 [C_20_H_31_CaN_6_O_12_S_2_]^+^) complex decomposes giving a remarkably simple spectrum; it breaks down releasing *Glu* (m/z 522.06 [CaC_15_H_24_N_5_S_2_O_5_]^+^), *Gly* (m/z 576.07 [CaC_18_H_26_N_5_S_2_O_10_]^+^), and OH (m/z 635.60 [CaC_20_H_31_N_6_S_2_O_11_]^+^) as neutrals. The further formation of the most informative calcium containing products of m/z 619.76, m/z 346.04, and m/z 379.07 (Figure 7B) is also observed. The breakage of CH_2_-S and S-S bonds leads to the formation of the ions of m/z 379 and 346, respectively. Thereafter, both calcium containing species decompose giving rise to low intensity ion series. Appearance of small mass calcium containing ions, in MS/MS spectrum of Ca-*GSSG* peptide complex, is additional evidence that calcium binding is *via* N terminus of the peptide. Therefore, *GSSG* involves calcium in an “open” type complex, in which the metal ion is not coordinated from both glutamic acids, assuming a behavior like a simple amino acid. Finally, the simplicity of MS/MS spectra indicates that the binding of Ca^2+^ ions to *GSH* and *GSSG* is to the deprotonated glutamyl carboxylic residue and to the NH amino function. Ca^2+^-peptide complexes undergo fragmentations that are determined by the location of the Ca binding site.

### Speciation in Biological Fluids

**FIGURE 8:**
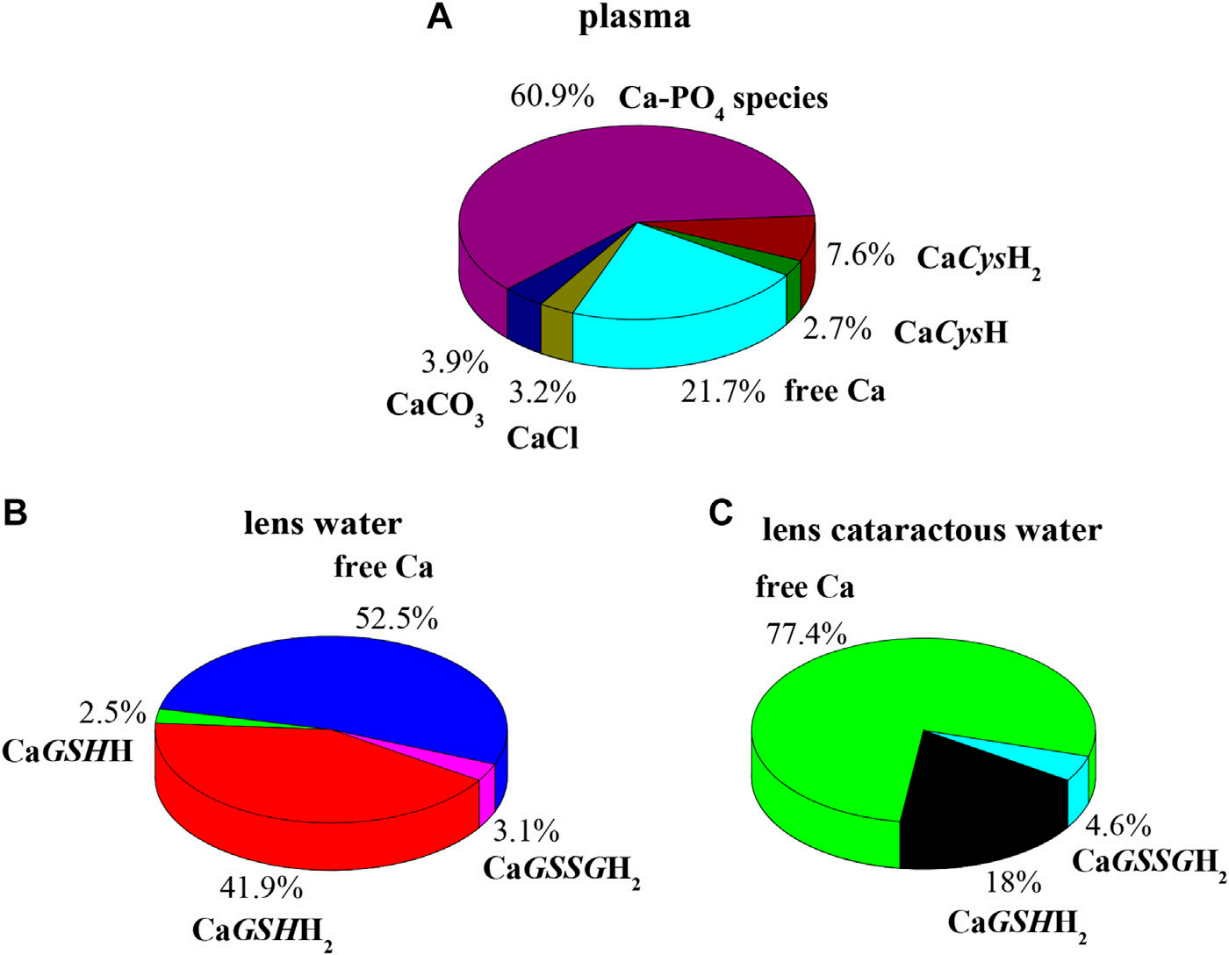
Ca-*Cys*, *GSH*, *GSSG* species in biological fluids at *t* = 37 °C, *I* = 0.15 mol L^−1^. **(A)** Plasma conditions (pH = 7.4); **(B)** lens water conditions (pH = 7.2); **(C)** lens cataractous water conditions (pH = 7.2).

In order to evaluate the relevance of the systems under study under real conditions, two biological fluids were considered. The first application consists in the evaluation of formation percentages of Ca^2+^ complex species, by considering plasma concentration, temperature, and ionic strength conditions (*t* = 37 °C, *I* = 0.15 mol L^−1^, C_Ca_ = 2.5 mmol L^−1^, C_Cys_ = 0.01 mmol L^−1^; C_GSH_ = 5.5 μmol L^−1^, C_GSSG_ = 0.5 μmol L^−1^, C_Cl_ = 0.1037 mol L^−1^, C_SO4_ = 0.49 mmol L^−1^, C_CO3_ = 24.9 mmol L^−1^, C_PO4_ = 1.6 mmol L^−1^) (Lentner, 1983). In these conditions, at pH = 7.4 the main species is CaPO_4_, with a percentage of 60.9%. The most important species among ones under study are Ca*Cys*H_2_ and Ca*Cys*H, although their sum just reaches 10.3%, as shown in Figure 8A.

The second application is based on lens aqueous solution. In the human eye the aqueous humor is located between the lens and the cornea. It is a gelatinous fluid where antioxidants, such as *GSH* and *Cys*, were investigated widely, since they serve as markers for eye diseases and infections. In the lens, the antioxidant *GSH* and ascorbic acid have unusually high concentration (Pescosolido et al., 2016). The functions performed by *GSH* with ascorbic acid in the lens are manifold. Among them, very important is the protection of protein thiol groups against oxidation agents and the detoxification of hydrophobic species in reactions catalyzed by glutathione S-transferase enzymes. In cataractous lens as well as in the aging lens, calcium concentration increases, and destruction of ascorbic acid and reduction of *GSH* content also occur (Chandorkar et al., 1980; Pescosolido et al., 2016). Accordingly, two different simulations were performed considering the composition of electrolyte and biological ligands in normal and in cataractous lens water. The obtained results are very different. Figure 8B represents the pie plot of Ca^2+^ complex species at pH = 7.2, by considering normal lens water concentrations (C_Ca_ = 0.01 mmol L^−1^, C_Cys_ = 0.0143 mmol L^−1^; C_GSH_ = 3.28 mmol L^−1^, C_GSSG_ = 0.095 mmol L^−1^, C_Cl_ = 0.79 mmol L^−1^, C_Ascorbic Acid_ = 1 mmol L^−1^) (Chandorkar et al., 1980; Königsberger et al., 2015). In this case, among species formed by Ca^2+^-ligands under study, those containing *GSH* form with higher percentages with a sum of 44.4%. The results significantly change by considering concentrations in cataractous lens water. Several studies reported that the level of reduced *GSH* in the lens decreases with the development of cataract (Kisic et al., 2012; Pescosolido et al., 2016). In this way over the years, *GSH* content reduces up to 73%, and *GSSG* content levels increase up to 18% (Pescosolido et al., 2016). Accordingly, the pie plot at pH = 7.2, under cataractous lens water conditions, was depicted in Figure 8C (C_Ca_ = 0.12 mmol L^−1^, C_Cys_ = 0.0143 mmol L^−1^; C_GSH_ = 0.9 mmol L^−1^, C_GSSG_ = 0.11 mmol L^−1^, C_Cl_ = 0.43 mmol L^−1^) (Chandorkar et al., 1980; Kisic et al., 2012; Königsberger et al., 2015; Pescosolido et al., 2016). In this case, percentage of Ca*GSH*H_2_ species drastically decreases while remaining significant (from 41.9 to 18%); Ca*GSSG*H_2_ increases slightly while resulting in an irrelevant species. These simulations confirm the need of knowledge of reliable formation constants at different conditions to predict the relevance of the species in real systems.

### Dependence of Formation Constants on the Temperature

Formation constant values of the complex species reported in Table 2, obtained by potentiometric measurements at *t* = 15, 25, 37 °C, were analyzed for the determination of the formation enthalpy changes of the species, *via* the van't Hoff equation, already employed for several other systems (Cardiano et al., 2019; Cordaro et al., 2019; Foti and Giuffre, 2020; Giuffrè et al., 2019; Giuffrè et al., 2020):

$${\text{logβ}}_{\text{T}}{=\text{logβ}}_{\text{θ}}{+\text{ΔH}}^{\text{0}}\left(1/\text{θ}-1/T\right)\;\text{Rln}10,$$ (3)

where log*β*_T_ is the formation constant at a specific ionic strength and temperature (expressed in Kelvin), log*β*_θ_ is the formation constant at *T* = 298.15 K, and Δ*H* ^0^ is the formation enthalpy change at *T* = 298.15 K in kJ mol^−1^, R = 8.314,472 J K^−1^ mol^−1^.

**FIGURE 9:**
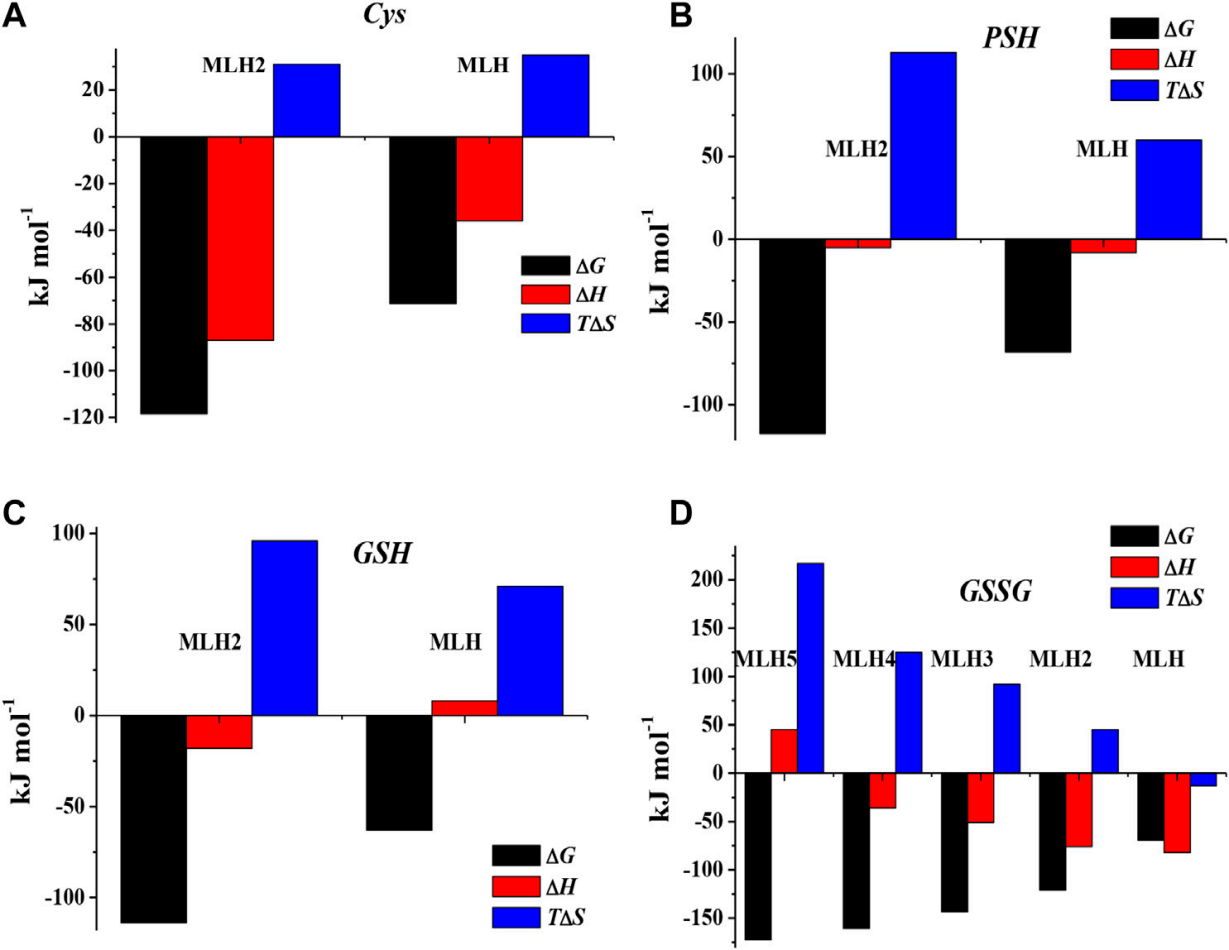
Bar plot of Δ*G*, Δ*H*, and *T*Δ*S* referring to Ca^2+^-*Cys* **(A)**, Ca^2+^-*PSH* **(B)**, Ca^2+^-*GSH* **(C)**, and Ca^2+^-*GSSG* **(D)** species at *t* = 25 °C, *I* = 0.15 mol L^−1^ in NaCl, according to overall formation reaction.

The values of formation enthalpy changes of all the species of Ca^2+^-*Cys*, -*PSH*, -*GSH*, and -*GSSG* systems are collected in Table 5, together with entropy and free energy values. They are also shown as bar plot in Figure 9, to better highlight the contribution to the formation free energy of the enthalpy and entropy thermodynamic parameters. Since the interactions between Ca^2+^ and the ligands understudy are mainly of electrostatic nature, it is expected that the entropic term gives the highest contribution to the free energy change, due to the orientation disorder given by the solvation water molecules. This was found for most species (except for MLH one formed by the interaction with *Cys* ligand, MLH_2_, and MLH ones containing *GSSG* ligand).

**TABLE 5:** Thermodynamic formation parameters of Ca^2+^-*Cys*, -*PSH*, -*GSH*, -*GSSG* species at *t* = 25 °C, *I* = 0.15 mol L^−1^ in NaCl.

Ligand	Species	−Δ*G* ^a^ ^b^	Δ*H* ^a^ ^b^	*T*Δ*S* ^a^ ^b^
*Cys*	MLH_2_	118.5	−87 (2) ^c^	31
	MLH	71.3	−36 (3)	35
*PSH*	MLH_2_	117.6	−5 (8)	113
	MLH	68.2	−8 (8)	60
*GSH*	MLH_2_	114.0	−18 (12)	96
	MLH	62.9	8 (18)	71
*GSSG*	MLH_5_	172.3	45 (12)	217
	MLH_4_	160.7	−36 (16)	125
	MLH_3_	143.4	−51 (12)	92
	MLH_2_	121.0	−76 (10)	45
	MLH	69.4	−82 (9)	−13

^a^ Referring to overall formation constants.

^b^ Expressed in kJ mol^−1^.

^c^ ≥95% of confidence interval.

### Sequestering Ability

The sequestering capacity represents the tendency, in solution, of a ligand to complex metal cation forming metal-ligand species, which allow for reducing the concentration of the free metal cation in solution. The stability of the complex species formed in solution influences the concentration of the free metal ion. The higher the stability of the formed species, the lower the concentration of the free cation. Considering the whole pH range, different metal-ligand species are formed in solution; each of them contributes to the sequestration of the metal cation. In order to describe the sequestering capacity of a given ligand with respect to a metal cation, it is not enough to know the formation constant values and the formation percentages of the different metal-ligand species. It is necessary to consider that different metal-ligand systems, having formation constants different from each other, can show the same formation percentages at a given pH and vice versa. Furthermore, all the equilibria in which the ligand and the metal ion under study take part must be considered, namely, ligand protonation, metal ion hydrolysis reactions, and weak interactions with the background salt. For these reasons, an empirical parameter, pL_0.5_, was proposed, which represents the cologarithm of the ligand concentration necessary to sequester 50% of the metal cation present in traces. The traces are precisely the concentration conditions with which many metal cations are present in natural fluids. To evaluate for quantitative purposes the sequestering capacity of a ligand with respect to a metal cation, the following Boltzmann-type equation with asymptotes 0 for pL→ 0 and 1 for pL→∞ was used (Gianguzza et al., 2012; Falcone et al., 2013; De Stefano et al., 2016):

$$\chi =\frac{1}{1+10^{\left(\text{pL}-{\text{pL}}_{0.5}\right)}},$$ (4)

where χ is the sum of the molar fractions of the metal-ligand species and pL is the cologarithm of the total ligand concentration. This parameter depends on system conditions, such as temperature, pH, and ionic strength.

**FIGURE 10:**
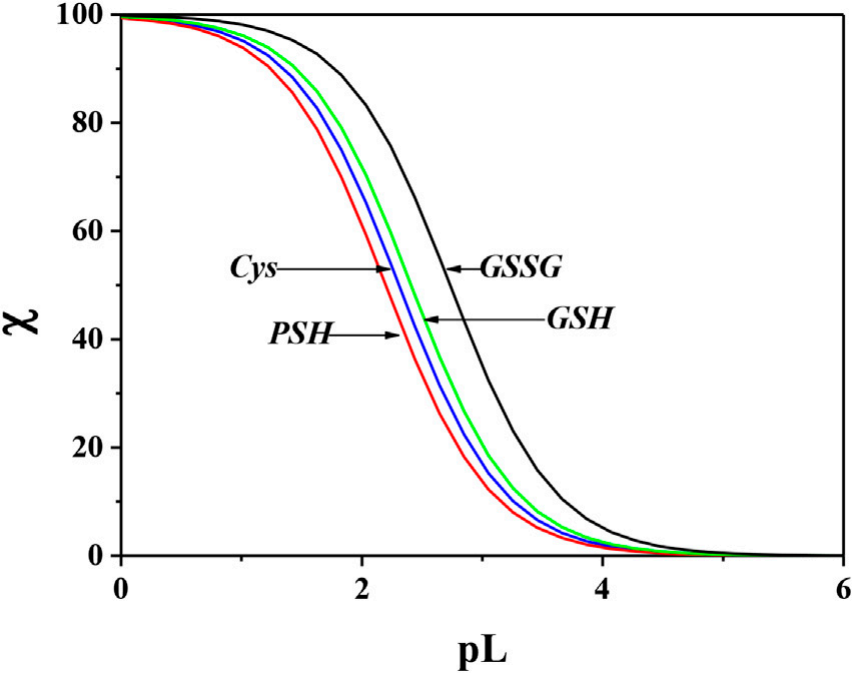
Comparison of sequestering ability of *Cys*, *PSH*, *GSH*, and *GSSG* toward Ca^2+^ under physiological conditions (pH = 7.4, *t* = 37 °C, *I* = 0.15 mol L^−1^).

In order to evaluate the sequestering capacity of *Cys*, *PSH*, *GSH*, and *GSSG* ligands toward Ca^2+^, pL_0.5_ values at different pH and temperatures were calculated. The results obtained are reported in Supplementary Table S5. Figure 10 illustrates the sequestering capacity of *Cys*, *PSH*, *GSH*, and *GSSG* ligands toward Ca^2+^ under physiological conditions (pH = 7.4, *t* = 37 °C, *I* = 0.15 mol L^−1^). As can be seen, the sequestering capacities of the ligands toward Ca^2+^ under physiological conditions follow the order:

$${\text{pL}}_{\text{0}\text{.}5}\left(\text{GSSG}\right){\text{>pL}}_{\text{0}\text{.}5}\left(\text{GSH}\right){\text{>pL}}_{\text{0}\text{.}5}\left(\text{Cys}\right){\text{>pL}}_{\text{0}\text{.}5}\left(\text{PSH}\right)$$

By comparing these data with those relating to the stepwise formation constants of Ca^2+^-ligand species, obtained by potentiometric measurements under physiological conditions, it is possible to find a different order of stability for the MLH_2_ species:

log⁡K(PSH)>log⁡K(GSSG)>log⁡K(GSH)>log⁡K(Cys)

and a further different order for the MLH species:

log⁡K(PSH)>log⁡K(GSH)>log⁡K(GSSG)≈log⁡K(Cys)

This underlines the importance of calculating the sequestering ability that, taking into account all the interactions, can be different with respect to the order of stability assessed for a single species and reveal the “real” trend of the ligands.

### Literature Comparisons

In literature databases there are few thermodynamic data on interactions of ligands under study with Ca^2+^ (Martell et al., 2004; May and Murray, 2001; Pettit and Powell, 2001). As regards Ca^2+^-*Cys* system, a paper reports at *t* = 25 °C and *I* = 0.1 mol L^−1^ logβ = 1.92 for ML species and several ternary species with other ligands (Ramamoorthy and Manning, 1975). This only value cannot be compared with the results with this paper, since the speciation model is totally different. In the case of Ca^2+^-*GSH* system, a speciation model at *t* = 37 °C and *I* = 0.15 mol L^−1^ with four species, namely, MLH_2_, MLH, ML, and MLOH, with logβ = 20.68, 12.89, 3.84, -6.46, respectively, is reported (Touche and Williams, 1976). These values can be compared with ours, as regards the common species, *i.e*., MLH_2_ and MLH, in the same experimental conditions (logβ = 20.14, 11.66, respectively). The significant differences probably can be attributed to the different speciation model considered. In a paper of Singh, where formation constant values of *GSH* with several metal cations, namely, Ca^2+^, Mg^2+^, Cu^2+^, Pb^2+^, Ni^2+^, Zn^2+^, Co^2+^, Cd^2+^, and Mn^2+^, are reported, only one formation constant value referred to ML species was obtained for each system, including one containing Ca^2+^ (Singh et al., 2001). For this reason, this formation constant value cannot be compared with results here reported.

In a more recent paper, a fairly similar speciation model with three species was found, namely, MLH_2_, MLH, and ML, where logβ = 19.27, 11.08, 1.60, respectively (*t* = 25 °C, *I* = 0.15 mol L^−1^) (Cigala et al., 2012). In this paper, the values obtained under the same conditions for MLH_2_ and ML species are logβ = 20.39, 11.53, respectively. The agreement in this case, mainly for MLH species, is quite satisfactory.

## Conclusion

The main purpose of this study was obtaining consistent speciation models and reliable thermodynamic data referring to Ca^2+^-bioligands systems, based on the results gained *via* different analytical techniques. Speciation models and stability formation constants obtained by potentiometry were confirmed by ^1^H NMR spectroscopy. Indeed, the comparative analysis of the chemical shift values of the studied bioligands allows for reasonably affirming that all of them act as chelating agents of Ca^2+^. MALDI MS confirmed the formation of complexes and MS/MS experiments and, moreover, indicated different complexing behaviors of the ligands toward Ca^2+^. The results suggest that *Cys* and *PSH* act as bidentate ligands giving rise to six-membered cycles *via* O and S; *GSH* and *GSSG* bind to Ca^2+^ ion *via* O and N. By potentiometry, formation constant values under different temperatures were evaluated. In this way were also obtained *T*Δ*S* and Δ*H* values, necessary to calculate formation constants at different temperatures. The sequestering ability of *Cys*, *PSH*, *GSH*, and *GSSG* toward Ca^2+^ was evaluated under different pH and temperature conditions, with particular attention to those simulating biological fluids, evidencing an interesting trend.

Finally, obtained stability data were crucial to gain simulations under biological fluid conditions, as blood and lens water, and pointed out the importance of reliable thermodynamic data for simulations useful for applications to real systems, characterized by variable composition and pH.

## Data Availability

The original contributions presented in the study are included in the article/Supplementary Material; further inquiries can be directed to the corresponding author.
