# Supplementary material for: Ca2+ Complexation With Relevant Bioligands in Aqueous Solution: A Speciation Study With Implications for Biological Fluids
Source: Front Chem. 2021 Feb 24;9:640219. doi: 10.3389/fchem.2021.640219 (PMC7953420; doi:10.3389/fchem.2021.640219)
Supplement: Supplementary file 1 [file table1.docx]

**Supplementary Information**

**Supplementary Table S1.** Hydrolysis Constants of Ca^2+^ at *I* = 0.15 mol L^-1^ (NaCl) and different temperatures

|  |  | logβ ^a)^ |  | Ref. |
| --- | --- | --- | --- | --- |
| Species | *t* = 15°C | *t* = 25°C | *t* = 37°C |  |
| CaOH^+^ | -13.14 | -12.87 | -12.56 | ^b)^ |

^a)^ Refer to the reaction: Ca^2+^ + H_2_O = CaOH^+^ + H^+^; ^b)^ Crea, F.; De Stefano, C., Milea, D., Pettignano, A., Sammartano, S. (2015). SALMO and S3M: A Saliva Model and a Single Saliva Salt Model for Equilibrium Studies, Bioinorg. Chem. Appl. 2015.

Supplementary Table S2. Protonation constants of *Cys*, *PSH, GSH, GSSG* at different temperatures, *I* = 0.15 mol L^-1^ in NaCl

| Ligand | Species ^a)^ |  | logβ^H^ |  | Ref. |
| --- | --- | --- | --- | --- | --- |
|  |  | *t* = 15°C | *t* = 25°C | *t* = 37°C |  |
| *Cys* | LH | 10.415 | 10.160 | 9.875 | ^b)^ |
|  | LH_2_ | 18.769 | 18.301 | 17.779 |  |
|  | LH_3_ | 20.817 | 20.325 | 19.776 |  |
|  |  |  |  |  |  |
| *PSH* | LH | 10.77 | 10.56 | 10.33 | ^c)^ |
|  | LH_2_ | 18.82 | 18.44 | 18.02 |  |
|  | LH_3_ | 20.67 | 20.27 | 19.83 |  |
|  |  |  |  |  |  |
| *GSH* | LH | 9.722 | 9.447 | 9.248 | ^c)^ |
|  | LH_2_ | 18.62 | 18.08 | 17.73 |  |
|  | LH_3_ | 22.20 | 21.567 | 21.26 |  |
|  | LH_4_ | 24.36 | 23.94 | 23.48 |  |
|  |  |  |  |  |  |
| *GSSG* | LH | 9.859 | 9.618 | 9.349 | ^d)^ |
|  | LH_2_ | 18.921 | 18.442 | 17.908 |  |
|  | LH_3_ | 22.689 | 22.212 | 21.680 |  |
|  | LH_4_ | 25.802 | 25.319 | 24.781 |  |
|  | LH_5_ | 27.984 | 27.467 | 26.891 |  |
|  | LH_6_ | 29.486 | 28.987 | 28.370 |  |

^a)^ On the basis of reaction: L^z-^ + iH^+^ = LH_i_ ^(z-i)-^, charges omitted for simplicity; ^b)^ Cardiano, P., De Stefano, C., Giuffrè, O. and Sammartano, S. (2008). Thermodynamic and spectroscopic study for the interaction of dimethyltin(IV) with L-cysteine in aqueous solution. Biophys. Chem. 133, 19-27. ^c)^ Falcone, G., Foti, C., Gianguzza, A., Giuffrè, O., Napoli, A., Pettignano, A. and Piazzese, D. (2013). Sequestering ability of some chelating agents towards methylmercury(II). Anal. Bioanal. Chem. 405, 881-893. ^d)^ Crea, P., De Stefano, C., Kambarami, M., Millero, F.J. and Sharma, V.K. (2008). Effect of ionic strength and temperature on the protonation of oxidized glutathione. J. Sol. Chem. 37, 1245-1259.

**Supplementary Table S3.** Chemical shift (in ppm) of individual nuclei of Ca^2+^-ligand species at *t* = 25°C and *I* = 0.15 mol L^-1^ in NaCl

|  | **Ligand** | **MLH_5_** | **MLH_4_** | **MLH_3_** | **MLH_2_** | **MLH** |
| --- | --- | --- | --- | --- | --- | --- |
| **δ_CH_** | *Cys* | ⎯ | ⎯ | ⎯ | 3.96(2)^a)^ | 3.46(2)^a)^ |
| **δ_CH2_** |  | ⎯ | ⎯ | ⎯ | 3.00(3) | 3.06(3) |
| **δ_CH_** | *PSH* | ⎯ | ⎯ | ⎯ | 4.04(1)^a)^ | 3.69(1)^a)^ |
| **δ_CH3-a_** |  | ⎯ | ⎯ | ⎯ | 1.52(1) | 1.49(1) |
| **δ_CH3-b_** |  | ⎯ | ⎯ | ⎯ | 1.44(2) | 1.25(2) |
| **δ_CysCHα_** | *GSH* | ⎯ | ⎯ | ⎯ | 4.5(1)^a)^ | 3.6(1) ^a)^ |
| **δ_GlyCH2α_** |  | ⎯ | ⎯ | ⎯ | 3.73(1) | 3.65(1) |
| **δ_GluCHα_** |  | ⎯ | ⎯ | ⎯ | 3.7(1) | 2.9(1) |
| **δ_CysCH2β_** |  | ⎯ | ⎯ | ⎯ | 2.9(2) | 2.7(2) |
| **δ_GluCH2γ_** |  | ⎯ | ⎯ | ⎯ | 2.49(1) | 2.33(1) |
| **δ_GluCH2β_** |  | ⎯ | ⎯ | ⎯ | 2.11(7) | 1.55(7) |
| **δ*_Cys_*_CHα_** | *GSSG* | 4.01(7)^a)^ | 3.90 (7) ^a)^ | 3.84(7) ^a)^ | 3.72(7) ^a)^ | 3.72(7) ^a)^ |
| **δ_GlyCH3α_** |  | 4.12(3) | 3.67(3) | 3.77(3) | 3.71(3) | 3.18(3) |
| **δ_GluCHα_** |  | 3.21(2) | 3.23(2) | 3.23(2) | 3.25(2) | 3.26(2) |
| **δ*_Cys_*_CH3β_** |  | 2.94(1) | 2.92(1) | 2.92(1) | 2.92(1) | 2.90(1) |
| **δ_GluCH3γ_** |  | 2.57(2) | 2.46(2) | 2.49(2) | 2.48(2) | 2.33(2) |
| **δ_GluCH3β_** |  | 2.21(7) | 2.08(7) | 2.11(7) | 2.10(7) | 1.81(7) |

^a)^ ≥95% of confidence interval.

**Supplementary Table S4.** Chemical shift (in ppm) of individual nuclei of *GSSG* ligand species at *t* = 25°C and *I* = 0.15 mol L^-1^ in NaCl

|  | | **L** | | **LH** | **LH_2_** | **LH_3_** | | **LH_4_** | | **LH_5_** | | **LH_6_** |
| --- | --- | --- | --- | --- | --- | --- | --- | --- | --- | --- | --- | --- |
| **δ*_Cys_*_CHα_** | 3.71(1)^a)^ | | 3.72(1)^a)^ | | 3.71(1)^a)^ | 3.81(1)^a)^ | 3.94(1)^a)^ | | 3.98±0.01^a)^ | | 3.95±0.01^a)^ | |
| **δ*_Gly_*_CH2α_** | 3.23(1) | | 3.43(1) | | 3.72(1) | 3.73(1) | 3.75(1) | | 3.99±0.01 | | 4.04±0.01 | |
| **δ*_Glu_*_CHα_** | 3.25(1) | | 3.25(1) | | 3.25(1) | 3.24(1) | 3.23(1) | | 3.22±0.01 | | 3.21±0.01 | |
| **δ*_Cys_*_CH2β_** | 2.90(1) | | 2.90(1) | | 2.91(1) | 2.92(1) | 2.93±0.01 | | 2.94±0.01 | | 2.94±0.01 | |
| **δ*_Glu_*_CH2_γ** | 2.34(1) | | 2.39(1) | | 2.48(1) | 2.48(1) | 2.48±0.01 | | 2.54±0.01 | | 2.55±0.01 | |
| **δ*_Glu_*_CH2β_** | 1.84(2) | | 1.94(2) | | 2.10(2) | 2.10(2) | 2.11±0.02 | | 2.18±0.02 | | 2.18±0.02 | |
| **δ_NH_** | ⎯ | | ⎯ | | ⎯ | ⎯ | ⎯ | | ⎯ | | ⎯ | |
| **δ_NH’’_** | ⎯ | | ⎯ | | ⎯ | ⎯ | ⎯ | | ⎯ | | ⎯ | |

^a)^ ≥95% of confidence interval.

**Supplementary Table S5.** Sequestering ability of *Cys*, *PSH*, *GSH* e *GSSG* towards Ca^2+^ at different temperature and pH values, at *I* = 0.15 mol L^-1^ in NaCl

| Ligand | pH | *t* /°C | pL_0.5_ |
| --- | --- | --- | --- |
| *Cys* | 7.4 | 15 | 2.43 |
|  | 8.1 | 15 | 2.38 |
|  | 7.4 | 25 | 2.49 |
|  | 8.1 | 25 | 2.43 |
|  | 7.4 | 37 | 2.31 |
|  | 8.1 | 37 | 2.28 |
|  |  |  |  |
| *PSH* | 7.4 | 15 | 1.56 |
|  | 8.1 | 15 | 1.54 |
|  | 7.4 | 25 | 2.09 |
|  | 8.1 | 25 | 1.87 |
|  | 7.4 | 37 | 2.20 |
|  | 8.1 | 37 | 2.02 |
|  |  |  |  |
| *GSH* | 7.4 | 15 | 1.77 |
|  | 8.1 | 15 | 1.77 |
|  | 7.4 | 25 | 1.76 |
|  | 8.1 | 25 | 1.73 |
|  | 7.4 | 37 | 2.41 |
|  | 8.1 | 37 | 2.40 |
|  |  |  |  |
| *GSSG* | 7.4 | 15 | 2.72 |
|  | 8.1 | 15 | 2.73 |
|  | 7.4 | 25 | 2.74 |
|  | 8.1 | 25 | 2.72 |
|  | 7.4 | 37 | 2.73 |
|  | 8.1 | 37 | 2.66 |

| **** |  |
| --- | --- |
| **** | **** |

**Supplementary Figure S1.** Bar plot of Δ*G*, Δ*H*, *T*Δ*S* referring to Ca^2+^-*Cys*, -*PSH*, -*GSH*, -*GSSG* species at *t* = 25°C, *I* = 0.15 mol L^-1^ in NaCl, according to the stepwise formation reaction (2).


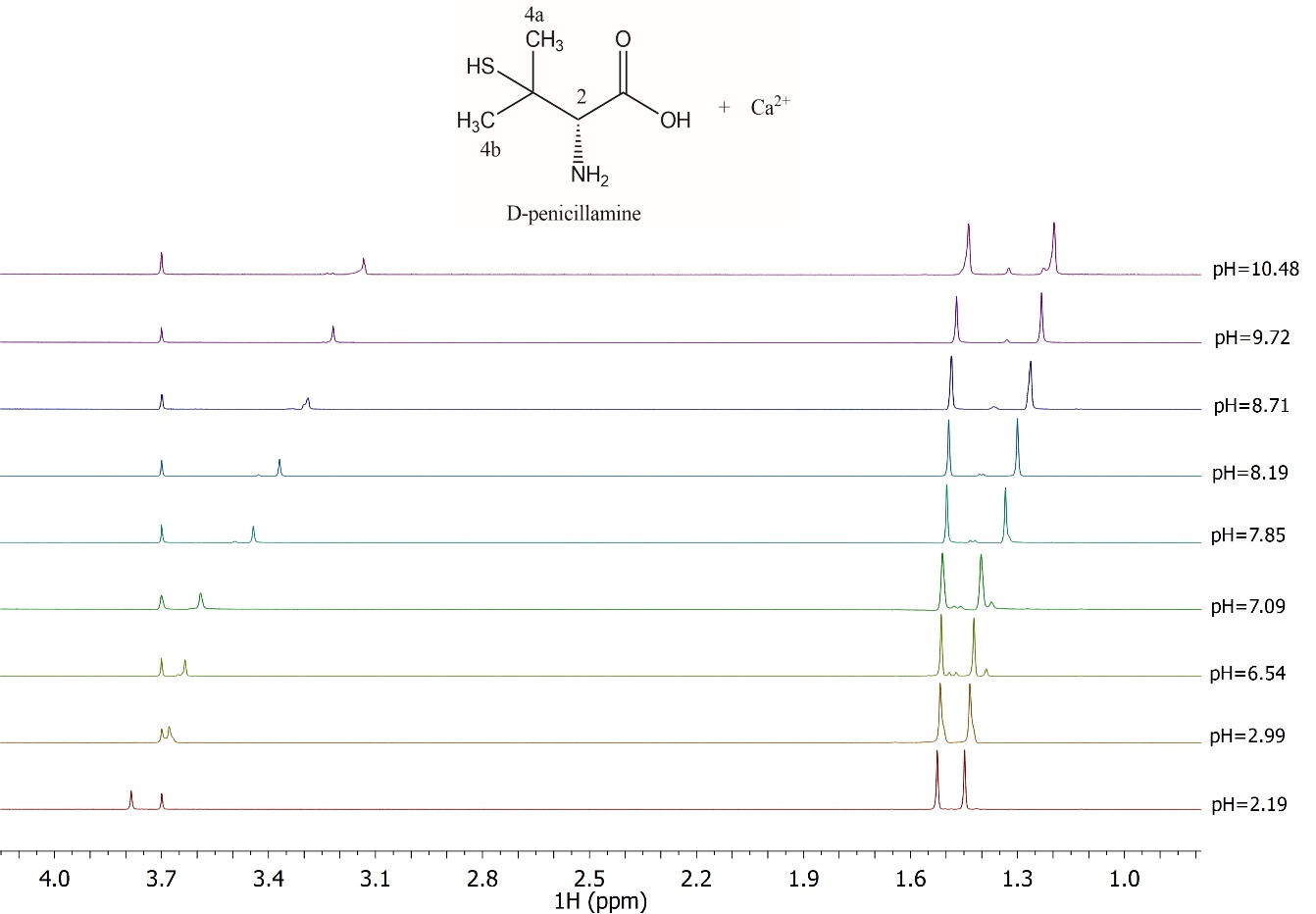


**Supplementary Figure S2.** ^1^H NMR spectra on solutions containing Ca^2+^ (M) and *PSH*(L) at C_M_ = 7.5 mmol L^-1^, C_L_ = 7.5 mmol L^-1^, *t* = 25°C, *I* = 0.15 mol L^-1^ in NaCl, 2.2 ≤ pH ≤ 10.5.

**
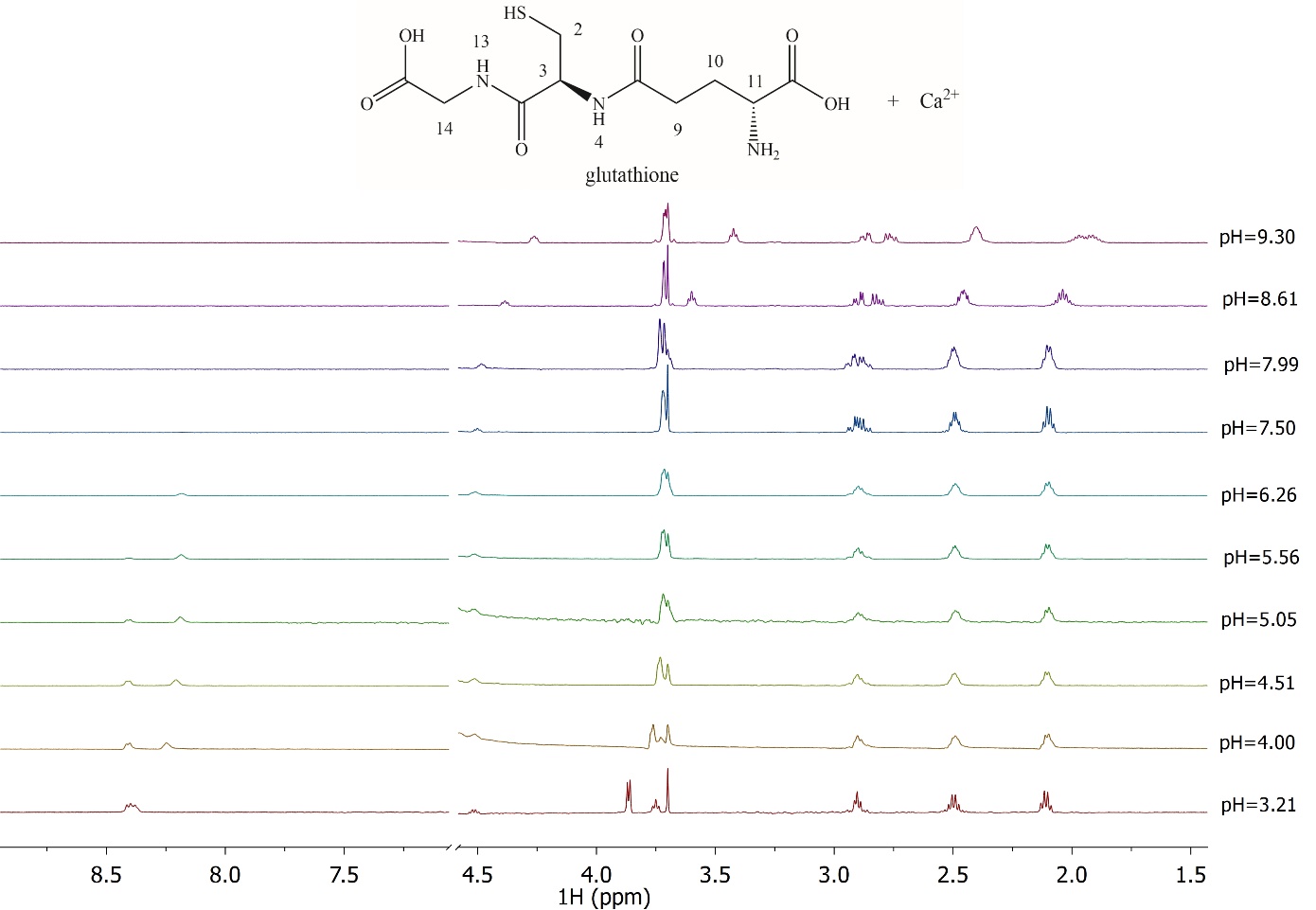
**

**Supplementary Figure S3.** ^1^H NMR spectra on solutions containing Ca^2+^ (M) and *GSH*(L) at C_M_ = 7 mmol L^-1^, C_L_ = 5 mmol L^-1^, *t* = 25°C, *I* = 0.15 mol L^-1^ in NaCl, 3.2 ≤ pH ≤ 9.3.


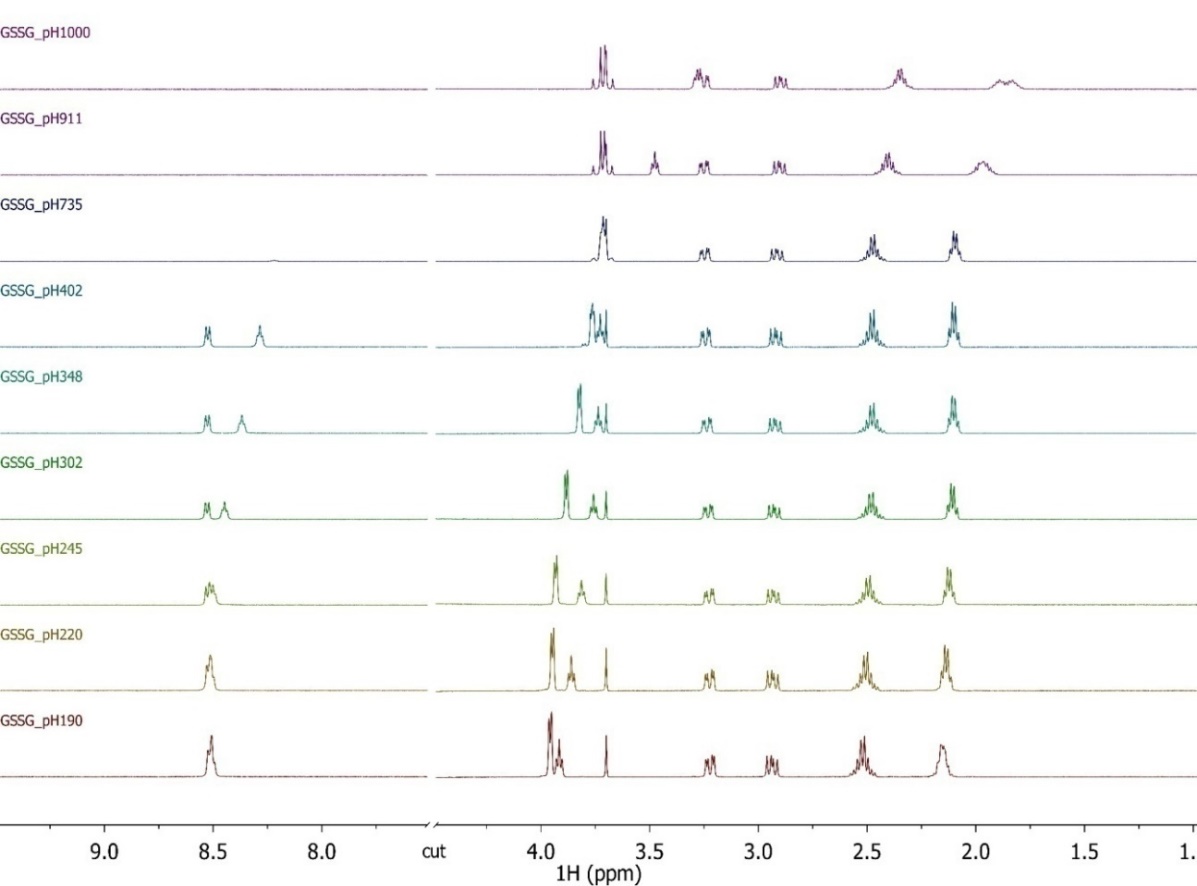

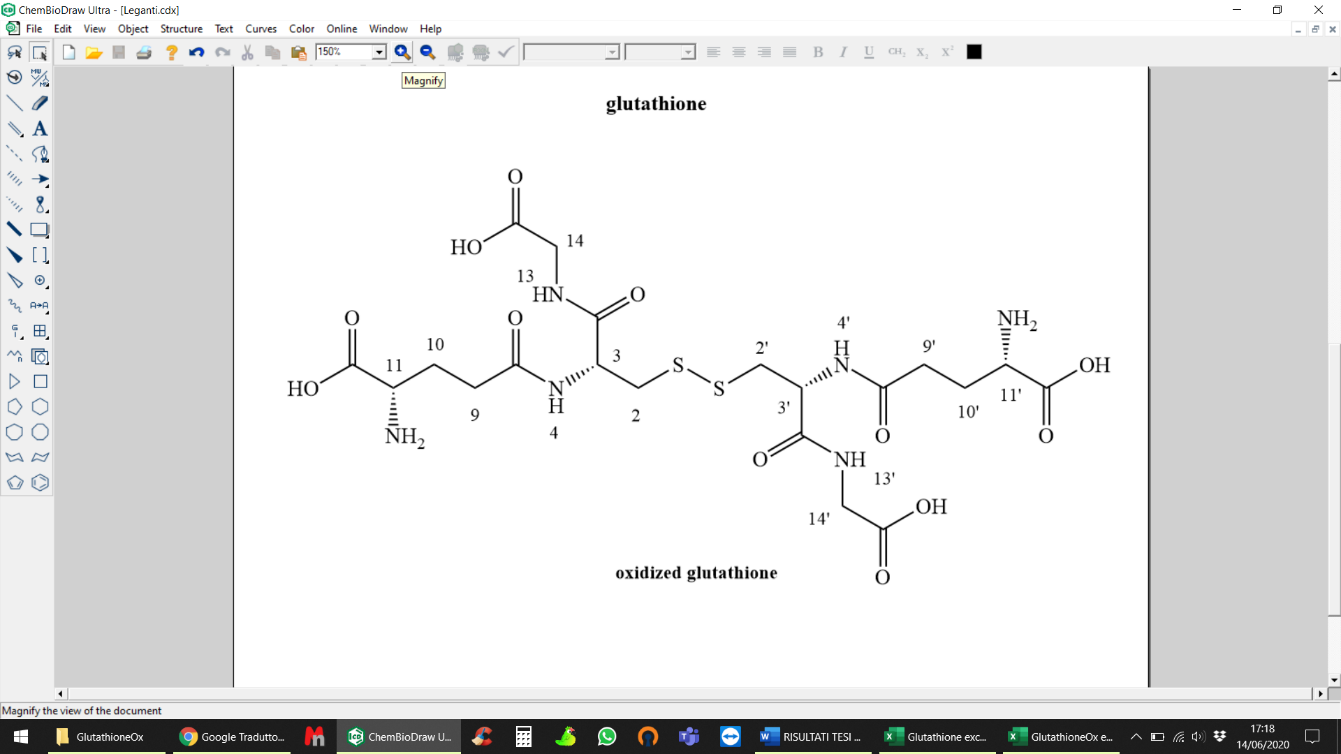


pH = 10.0

pH = 9.1

pH = 7.3

pH = 4.0

pH = 3.5

pH = 3.0

pH = 2.4

pH = 2.2

pH = 1.9

**Supplementary Figure S4.** ^1^H NMR spectra on solutions containing *GSSG* at C = 6 mmol L^-1^, *t* = 25°C, *I* = 0.15 mol L^-1^ in NaCl, 1.9 ≤ pH ≤ 10.0.
